# Modelling HIV and MTB Co-Infection Including Combined Treatment Strategies

**DOI:** 10.1371/journal.pone.0049492

**Published:** 2012-11-28

**Authors:** Santosh Ramkissoon, Henry G. Mwambi, Alan P. Matthews

**Affiliations:** 1 Physics-Durban Academic Group (School of Chemistry and Physics), University of KwaZulu-Natal, Westville Campus, Durban, South Africa; 2 School of Mathematics, Statistics and Computer Science, University of KwaZulu-Natal, Pietermaritzburg Campus, Pietermaritzburg, South Africa; Harvard School of Public Health, United States of America

## Abstract

A new host-pathogen model is described that simulates HIV-MTB co-infection and treatment, with the objective of testing treatment strategies. The model includes CD4+ and CD8+ T cells, resting and activated macrophages, HIV and *Mycobacterium tuberculosis* (MTB). For TB presentation at various stages of HIV disease in a co-infected individual, combined treatment strategies were tested with different relative timings of treatment for each infection. The stages were early HIV disease, late HIV disease and AIDS. The main strategies were TB treatment followed by anti-retroviral therapy (ART) after delays of 15 days, 2 months and 6 months. ART followed by TB treatment was an additional strategy that was tested. Treatment was simulated with and without drug interaction. Simulation results were that TB treatment first followed by ART after a stage-dependent delay has the best outcome. During early HIV disease a 6 month delay is acceptable. During late HIV disease, a 2 month delay is best. During AIDS it is better to start ART after 15 days. However, drug interaction works against the benefits of early ART. These results agree with expert reviews and clinical trials.

## Introduction

In many developing countries, most notably in Africa where the HIV epidemic is particularly severe, individuals infected with HIV (human immunodeficiency virus) are initiated on ART (anti-retroviral therapy) only when their CD4+ T cell count is below 200 per mm^3^. At this stage an HIV-infected individual is likely to be co-infected with *Mycobacterium tuberculosis* (MTB) due to a new MTB infection or latent MTB re-activated due to a weakened immune system. Although current therapies for HIV and TB are effective, there are several problems associated with their combination [1]–[6].

First, anti-retrovirals have significant side effects which are increased when combined with anti-TB drugs due to overlapping toxicity profiles. Severe side effects may compromise strict adherence to the drug regime resulting in sub-optimal treatment and development of drug-resistant strains of both MTB and HIV, and consequently accelerated progression of both diseases. Second, drug interaction may lead to diminished therapeutic results depending on the choice of drugs. Specifically, some anti-TB drugs reduce concentration of certain anti-retroviral drugs by as much as 90% [4]. Third, after ART the recovery of the immune system may result in Immune Reconstitution Inflammatory Syndrome (IRIS) which is especially problematic for an individual with TB.

Due to these problems the timing of ART relative to TB treatment for co-infection is an important question. As stated in 2010 by Abdool Karim et al. [7], “The optimal timing for the initiation of antiretroviral therapy in relation to tuberculosis therapy remains controversial.” Clinical trials to address this question have been reviewed by Piggott and Karakousis [6] and include the CAMELIA trial (Cambodian Early versus Late Introduction of Antiretroviral drugs) [8], another recent trial [9], and the CAPRISA (Centre for the AIDS Programme of Research in South Africa) SAPIT trial (Starting Antiretroviral therapy at 3 Points in TB) [5], [7]. SAPIT results [7] support concurrent treatment of HIV and TB for co-infected individuals who have CD4+ T cell counts <500 per mm^3^. SAPIT had a sequential arm (ART after TB treatment) and two integrated arms (earlier and later ART during TB treatment). The integrated arms proved far superior and the sequential arm had to be stopped. A further analysis [5] of earlier versus later ART in the integrated arms showed that overall there was little difference between outcomes, but individuals with a CD4 count <50 per mm^3^ benefited from earlier ART (initiated within 4 weeks of the start of TB treatment).

For many years mathematical and computational models have been applied to host-pathogen interaction dynamics and constitute a valuable means of analysis that complements clinical research [10]. Such models have been developed for various aspects of HIV infection [11]–[27] and for MTB infection [28]–[38]. Only a few [39]–[42] have been developed for HIV-MTB co-infection. Of the latter the earliest [40] has four populations: T cells (CD4+ and CD8+ combined), macrophages, HIV and MTB. Bauer et al. [39] have a far more complex MTB component that includes cytokines, similar to the approach in a number of other models [29], [30], [35]–[38]. The co-infection model of Magombedze et al. [42] which builds on their earlier work [18], [32–[34], [41] has an intermediate level of complexity with a similar set of populations to Bauer et al. [39] but no cytokines. Regarding modelling of treatment, standard TB treatment only is included in two models [33], [40] and ART only by a number of others [12], [15]–[Bibr pone.0049492-Kirschner1] [Bibr pone.0049492-Kirschner2] [18], [23], [25], [26]. Only Magombedze et al. [42] model both ART and TB treatment. They simulated three options: simultaneous treatment, ART only and TB treatment only, and concluded that simultaneous treatment has the best outcome.]

We present a new model that builds on previous work [16], [20], [26], [39], [40], [42] and includes elements of other models referenced when described. The biological processes involved in HIV and MTB infection and the response of the immune system to each have been described in various papers [22], [35], [39], [42] and are summarised in the model description in “Methods”. We include a process to simulate HIV disease progression and model different relative timings of treatment as done in clinical trials. For three timings of TB presentation (early HIV disease, late HIV disease, and AIDS) various treatment strategies are tested. The main strategies start with TB treatment and initiate ART after delays of 15 days, 2 months and 6 months. In addition, to test other possibilities, we include scenarios with ART first followed by TB treatment after 2 months and 6 months.

The overall aims of this paper are to present a new HIV-MTB co-infection model with treatment of both infections, applied to the question of optimal timing of ART relative to TB treatment. These aims have been achieved, firstly by the description and testing of a model that combines HIV and MTB infection, with treatment for both diseases, and secondly by the application of the model to test treatment strategies.

## Methods

### Model populations and outline

**Figure 1:**
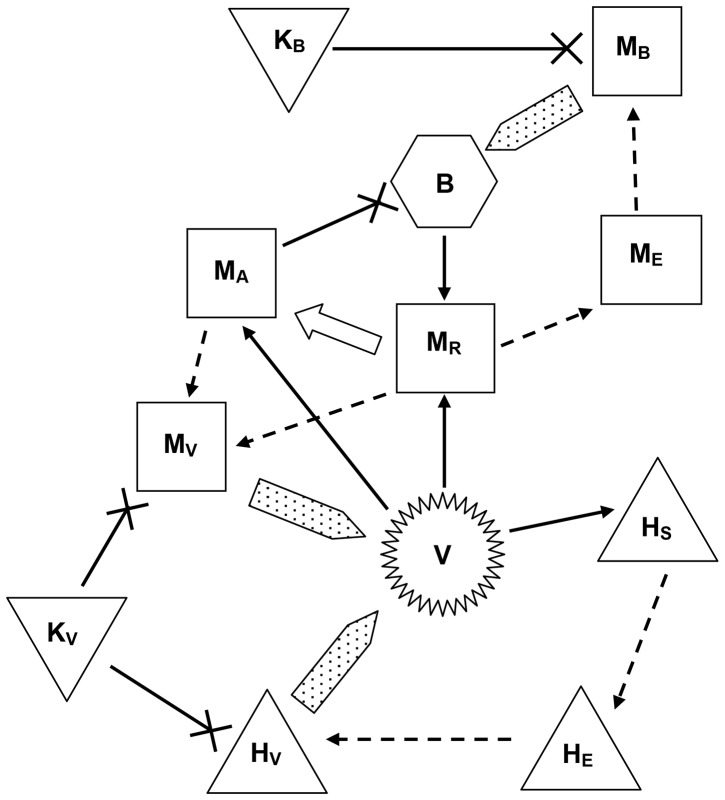
Flow diagram of the model. Populations are represented by shapes: CD4+ T cells by pyramids, CD8+ T cells by inverted pyramids, macrophages by squares, MTB by a hexagon and HIV by a jagged circle. Interactions are represented by arrows: infection by a solid arrow, transition by a dashed arrow, release of pathogen by a broad speckled arrow, activation by a broad white arrow and elimination by an arrow with a cross. HIV infects CD4+ T cells and both resting and activated macrophages. HIV-infected T cells and MTB-infected macrophages first enter an eclipse stage and then a productive stage when they release pathogen before dying. In the model, HIV-infected macrophages do not pass through an eclipse stage because their life-span is relatively long. Some resting macrophages ingest MTB and become infected, whereas other resting macrophages become activated and are able to phagocytose and eliminate MTB. CD8+ T cells eliminate infected cells.

The twelve populations of immune system cells and pathogens in the model are listed with initial values in Table 1 and their interactions are illustrated as a flow diagram in Figure 1. All populations depend on time *t* and are measured as number per mm^3^ (or microliter µL). The model is set in the lung compartment where alveolar macrophages and T cells interact with extracellular MTB (population *B*). HIV (*V*) is present in the blood which circulates constantly through the body and the lungs. Although HIV density varies with location in the body [13], we consider an average systemic viral load that is correlated with viral load in the peripheral blood. This viral load in turn is correlated with an average HIV infection rate of both CD4+ T cells and macrophages.

**Table 1:** Population variables and initial values.

Symbol	Description	Initial value (mm^−3^)
*H_S_*	Susceptible CD4+ T cells	1000
*H_E_*	HIV-infected T cells in eclipse stage	0
*H_V_*	HIV-infected T cells in productive stage	0
*K_V_*	HIV-specific CD8+ cytotoxic T cells	10
*K_B_*	MTB-specific CD8+ cytotoxic T cells	10
*M_R_*	Resting macrophages	200
*M_E_*	MTB-infected macrophages in eclipse stage	0
*M_B_*	MTB-infected macrophages in productive stage	0
*M_A_*	Activated macrophages	0
*M_V_*	HIV-infected macrophages	0
*V*	HIV virions	0.1
*B*	MTB bacteria	10

There are five varieties of T cells. The first are CD4+ helper T cells (*H_S_*), which are susceptible to HIV infection. When infected by HIV they are reclassified as eclipse-stage cells (*H_E_*) during the few days that HIV interacts with the cell structures in preparation for budding of new virions from the cell [11], [43]. This includes a biologically-realistic time delay in the infection-replication cycle. Once virion budding begins the eclipse-stage cells become productively infected cells (*H_V_*) that release virions during the one or two days before they die. In addition to death by HIV infection there is also death from viral proteins [44]. There are two small but variable populations of pathogen-specific CD8+ cytotoxic killer T cells (*K_V_* for HIV and *K_B_* for MTB) that proliferate in response to infected target cells, which they eliminate by lysis.

The macrophage population has five types. Resting macrophages (*M_R_*) first encounter MTB in the alveoli. The bacilli are ingested but not destroyed, and they replicate within the macrophages. The macrophages thus become MTB-infected, moving first into an eclipse stage (*M_E_*) during which bacteria are not released, and then into an infected stage (*M_B_*) during which bacteria are released by bursting of the macrophage due to a high bacterial load or by natural death of the macrophage. The presence of MTB stimulates recruitment of more macrophages from the blood, and generates an adaptive immune response in which *H_S_* cells activate resting macrophages to become activated macrophages (*M_A_*) that successfully phagocytose and destroy bacteria.

*H_S_* cells also stimulate proliferation of MTB-specific killer T cells that lyse *M_B_* cells. On lysis the bacterial load of the infected cell is released and may be phagocytosed. Furthermore, macrophages may be infected by HIV [39]–[Bibr pone.0049492-Kirschner4] [Bibr pone.0049492-Magombedze5] [42], [45], [46]. We consider only HIV infection of *M_R_* and *M_A_*, which become HIV-infected macrophages (*M_V_*). We do not consider HIV-MTB co-infection of macrophages [39] since that adds additional complexity for a small number of cells. However, we do include the effect of MTB infection increasing HIV production [39], [47] as one of the mutually aggravating features of co-infection.

### Modelling immune system collapse

It is difficult to model progression of HIV infection to AIDS because the mechanisms are not fully understood. Motivated by the approach of Kirschner and Webb [16] we simulate immune system collapse by assuming that the virion production rate of infected T cells and macrophages is increased over time by a factor *R_V_* due to mutations, increased efficiency in virion production, or the immune system's progressive inability to contain virion production [14], [40]. We first define *V_T_* as total viral burden since the start of infection, given as years of infection with reference viral load *V_ref_*, as
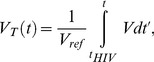
(1)where *t_HIV_* is the HIV infection date and time is in years. We then specify the replication increase factor *R_V_* in terms of a coefficient *p_V_* and a Weibull cumulative distribution function *Q*(*V_T_*) with scale parameter *σ* and shape parameter *k*:
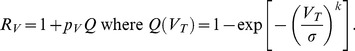
(2)Weibull functions are often used to model systems with failure times, and have been used for example to simulate progression to AIDS in epidemiological models at population level [48], [49].

HIV infection is understood to eventually exhaust the capacity of the body to produce CD4+ T cells [50], [51] and following but modifying the idea of Kirschner [40] this is modelled by a capacity reduction factor
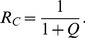
(3)This diminishes the source *s_H_* of CD4+ T cells by up to 50% as HIV infection progresses. Since in this model not all capacity is lost, an individual may experience a degree of immune reconstitution after ART that depends on degree of disease progression at the time of treatment.

### Modelling treatment

TB treatment is simulated by considering bacteriostatic and bactericidal drug action. Bacteriostatic drugs inhibit growth of MTB and are modelled by limiting bacterial growth by the parameter *ε_S_* which ranges from 0 to 1 with 1 corresponding to 100% effective. Bactericidal drugs kill MTB and are modelled by the parameter *ε_C_* in a bacterial death term.

Anti-retroviral therapy (ART) is simulated by considering both protease and reverse transcriptase inhibitors, and using effectiveness parameters [26], [42]. Protease inhibitors render HIV virions non-infectious, an action modelled with the parameter *ε_P_*. Reverse transcriptase inhibitors block HIV infection of T cells and macrophages, modelled with the parameter *ε_R_*. Both parameters range from 0 to 1, with 1 corresponding to 100% effective.

Interaction between anti-retrovirals (ARVs) and anti-TB drugs may reduce ARV efficacies [1]–[Bibr pone.0049492-Dean1] [Bibr pone.0049492-Kwara1] [4]. For example reduction of ARV absorption due to the commonly-used anti-TB drug rifampin varies from 30–90% for several protease inhibitors and from 20–96% for some reverse transcriptase inhibitors [4]. We do not attempt to model the various drug combinations, but as a simplification, in order to simulate a reasonable worst-case scenario, we use a single efficacy reduction parameter *d* that reduces both *ε_P_* and *ε_R_*. If *d* = 0 there is no drug interaction and full drug efficacy. ART is thus represented by a combined factor that reduces HIV infection, modified by drug interaction, given by
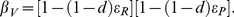
(4)


### Model equations

First we define functions that will simplify the population rate of change equations. Initial immune system response at the start of infection is simulated by multiplying several response terms by a phasing-in function *ρ_B,V_* (range 0–1, median *T_IR_*) that becomes non-zero after the infection date *t_INF_* where *INF* = *MTB* or *HIV*:
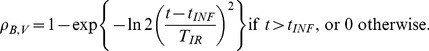
(5)The general health of the immune system is represented by
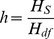
(6)where *H_df_* is the disease-free CD4+ T cell density (set at 1000 mm^−3^). We assume that several immune system responses are proportional to *h*. We model reduction of viral infection and replication rates due to non-cytotoxic action of *K_V_* [11], [42] by defining factors
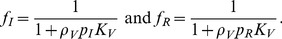
(7)Furthermore, HIV production is increased due to MTB infection [39], [47] by the factor
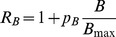
(8)where *B_max_* is a scale constant also used for setting a logistic limit to bacterial load.

Now we describe the population rate of change equations. The CD4+ helper T cell population is governed by
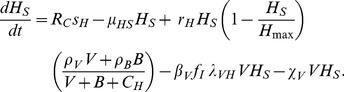
(9)The first term is the source, modified by the capacity reduction factor *R_C_*, and the second term is natural death. The third term is a proliferation response, where proliferation is proportional to a Michaelis-Menten function of both pathogens [40], and a logistic growth limit *H_max_* = 1500 is included [20] since the population does not grow without bound. The immune response factors *ρ_V_* and *ρ_B_* are attached separately to each pathogen. The fourth term is infection by HIV, reduced both by ART and CD8+ T cell non-cytotoxic action. The final term is killing of CD4+ T cells by toxic HIV proteins [44], assumed to be proportional to viral load. The eclipse-stage CD4+ T cells are governed by an equation with a gain term for infection and a loss term for transition to the productively infected stage:
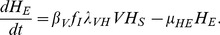
(10)The equation for productively infected T cells is
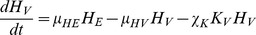
(11)where the first term is gain from the eclipse stage, the second is death due to budding of virions, and the last is loss due to lysis by *K_V_*. The lysis term could in principle have a Michaelis-Menten factor with half-saturation constant *C_L_* but the simpler form is suitable since *H_V_*≪*C_L_* (see “Parameters”). The term with the Michaelis-Menten factor is 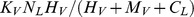 which is approximately 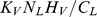, where *N_L_* is the maximum number of infected cells lysed per day by one CD8+ T cell. Then the lysis rate is given by
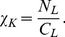
(12)The CD8+ T cells, HIV-specific and MTB-specific respectively, are governed by the following equations:
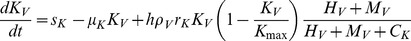
(13)and
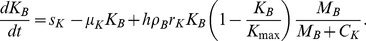
(14)Each equation has a source term, a natural death term, and a proliferation term similar to that of Magombedze et al. [42] but with a Michaelis-Menten function of infected cells. As for *H_S_* a logistic limit factor is included with a population upper limit *K_max_* because the populations do not grow without bound.

For resting macrophages, the recruitment term is given first as
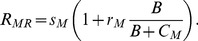
(15)The macrophage level is increased by attraction of macrophages to the infection site [47]. In several recent models [29], [35], [36], [38] recruitment depends on the level of MTB via the levels of activated and infected macrophages. Here, the disease-free source *s_M_* is amplified by a Michaelis-Menten function of bacteria (*B*). The resting macrophage evolution equation is then
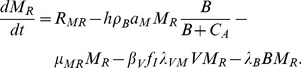
(16)Term 1 is recruitment. Term 2 is loss due to activation proportional to an activation coefficient *a_M_*, triggered by *B*, and helped by CD4+ T cells. Term 3 is natural death. Term 4 is HIV infection with a macrophage-specific infection rate *λ_VM_* and the same ART and CD8+ reduction factors as for T cell infection. Term 5 is MTB infection, with rate *λ_B_* related to three other model parameters by
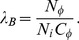
(17)The rationale is that each macrophage ingests on average 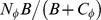 bacilli day^−1^, up to a maximum of *N_φ_* In the scenarios that will be simulated, *B* is well below the half-saturation level *C_φ_* so the approximation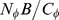 is acceptable (see “Parameters”). Then on average one macrophage is infected for every *N_i_* bacilli ingested.

Macrophages in the eclipse stage of MTB infection have an equation similar to Eq. (10) but with *B* as the pathogen:
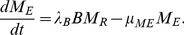
(18)The equation for MTB-infected macrophages has a form similar to Eq. (11):
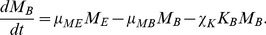
(19)Activated macrophages have the following equation, with the activation gain term from Eq. (16), a natural death loss term, and an HIV infection loss term:
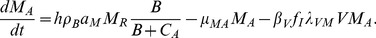
(20)HIV-infected macrophages have an equation of similar form to Eq. (11), with gain from HIV infection of both resting and active macrophages:
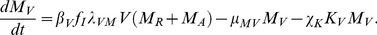
(21)HIV virions have an equation with a gain term due to viral production from HIV-infected T cells and macrophages (where *N_H,M_* are burst size per infected cell), and a loss term due to the combined effects of natural decay and clearance by neutralising antibodies:
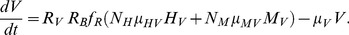
(22)The production term is increased by the disease progression factor *R_V_* and the MTB increase factor *R_B_*. It is decreased by the CD8+ T cell non-cytotoxic factor *f_R_*. The MTB population has a growth term due to intracellular replication given by
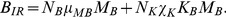
(23)The first term is release of *N_B_* bacilli per burst macrophage and the second term is release of *N_K_* bacilli per macrophage after lysis by a CD8+ T cell, where *N_K_*<*N_B_*. The MTB evolution equation is
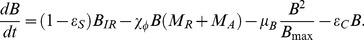
(24)Term 1 represents intracellular growth that is reduced with the bacteriostatic parameter *ε_S_*. Term 2 is loss due to phagocytosis by *M_R_* and *M_A_*. As described for Eq. (16), each macrophage ingests approximately 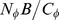 bacilli day^−1^ so the phagocytosis rate *χ_φ_* is related to other parameters by
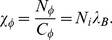
(25)Term 3 in Eq. (24) sets a logistic limit to bacterial level in infected but not yet necrotic tissue, and represents loss of bacteria by dissemination, flushing, or loss to necrotic regions. It has the same form as in Kirschner [40] and is useful in the model to prevent unbounded growth; high but limited growth will be sufficient to represent active infection that needs treatment. It has quadratic form so that it acts preferentially at high levels of *B* and exerts less influence at lower levels during development of infection. The final term is bactericidal drug action.

An analysis of the model is given in Appendix S1.

### Parameters

The model's parameters and their values are listed in Table 2, and are explained below.

**Table 2:** Parameters used in the simulations.

Symbol	Description	Value
*s_H_*	Source rate of CD4+ T cells *H_S_*	10 cells mm^−3^ day^−1^
*s_K_*	Source rate of CD8+ T cells *K_B,V_*	5 cells mm^−3^ day^−1^
*s_M_*	Source rate of resting macrophages *M_R_*	2 cells mm^−3^ day^−1^
*r_H_*	Proliferation response rate of CD4+ T cells *H_S_*	0.01 day^−1^
*r_K_*	Proliferation response rate of CD8+ T cells *K_B,V_*	1.5 day^−1^
*r_M_*	Macrophage recruitment factor	9
*a_M_*	Activation rate of macrophages	0.1 day^−1^
C_A_	Half-saturation constant for macrophage activation	500 bacilli mm^−3^
C_H_	Half-saturation constant for CD4+ T cell proliferation	1000 pathogen mm^−3^
*C_K_*	Half-saturation constant for CD8+ T cell response	2.5 cells mm^−3^
*C_M_*	Half-saturation constant for macrophage recruitment	500 bacilli mm^−3^
*C_L_*	Half-saturation constant for lysis	2500 cells mm^−3^
*C_φ_*	Half-saturation constant for phagocytosis	5000 bacilli mm^−3^
*μ_HS_*	Death rate of uninfected CD4+ T cells *H_S_*	0.01 day^−1^
*μ_HE_*	Transition rate of eclipse T cells *H_E_*	0.25 day^−1^
*μ_HV_*	Death rate of HIV-infected T cells *H_V_*	0.5 day^−1^
*μ_K_*	Death rate of CD8+ T cells *K_B,V_*	0.5 day^−1^
*μ_MR_*	Death rate of resting macrophages *M_R_*	0.01 day^−1^
*μ_ME_*	Transition rate of eclipse macrophages *M_E_*	0.05 day^−1^
*μ_MB_*	Burst rate of MTB-infected macrophages *M_B_*	0.1 day^−1^
*μ_MA_*	Death rate of activated macrophages *M_A_*	0.1 day^−1^
*μ_MV_*	Death rate of HIV-infected macrophages *M_V_*	0.01 day^−1^
*μ_V_*	Decay and clearance rate of HIV virions *V*	4 day^−1^
*μ_B_*	Loss rate of MTB *B*	0.1 day^−1^
*λ_VH_*	HIV infection rate of CD4+ T cells *H_S_*	10^−4^ virion^−1^ day^−1^
*λ_VM_*	HIV infection rate of macrophages *M_R_, M_A_*	10^−5^ virion^−1^ day^−1^
*λ_B_*	MTB infection rate of *M_R_*	8×10^−5^ bacillus^−1^ day^−1^
*N_H_*	HIV burst from HIV-infected T cell *H_V_*	1000 virions cell^−1^
*N_M_*	HIV burst from HIV-infected macrophage *M_V_*	800 virions cell^−1^
*N_φ_*	Maximum number of bacilli phagocytosed per *M_A,R_*	10 bacilli M_φ_^−1^ day^−1^ a
*N_i_*	Number of ingested bacilli per MTB infection	25 bacilli
*N_K_*	Number of bacilli released by lysis of *M_B_* by *K_B_*	35 bacilli cell^−1^
*N_B_*	Number of bacilli released by burst of *M_B_*	50 bacilli cell^−1^
*N_L_*	Maximum cells lysed by one CD8+ T cell *K_B.V_*	50 cells CD8^−1^ day^−1^
*χ_φ_*	Phagocytosis rate of *B* by *M_A,R_*	0.002 M_φ_^−1^ day^−1^ a
*χ_K_*	CD8+ T cell lysis rate	0.02 CD8^−1^ day^−1^
*χ_V_*	Death rate of CD4+ T cells *H_S_* due to viral proteins	10^−4^ virion^−1^ day^−1^
*p_I_*	HIV infection reduction coefficient	0.1 (cell/mm^3^)^−1^
*p_R_*	HIV replication reduction coefficient	0.1 (cell/mm^3^)^−1^
*p_V_*	HIV replication increase parameter	5
*p_B_*	MTB-induced HIV replication increase factor	0.2
*H_df_*	Disease-free CD4+ T cell level	1000 cells mm^−3^
*H_max_*	Maximum CD4+ T cell level	1500 cells mm^−3^
*K_df_*	Disease-free CD8+ T cell level	10 cells mm^−3^
*K_max_*	Maximum CD8+ T cell level	100 cells mm^−3^
*M_df_*	Disease-free macrophage level	200 cells mm^−3^
*B_max_*	Scale constant of MTB	1000 bacilli mm^−3^
*B_cure_*	MTB threshold for TB cure	1 bacillus mm^−3^
*V_ref_*	Viral load reference level	100 virions mm^−3^
*V_low_*	Undetectable viral load threshold	0.05 virions mm^−3^
*T_IR_*	Time for half-saturation of immune response	14 days
*T_median_*	Median of Weibull function for *V* increase	10 years
*T_width_*	Width (10%–90%) of Weibull function	10 years
*σ*	Scale parameter of Weibull function	11.362 years
*k*	Shape parameter of Weibull function	2.871
*ε_P_*	Protease inhibition drug efficacy	0 or 0.75^b^
*ε_R_*	Reverse transcriptase inhibition drug efficacy	0 or 0.75^b^
*ε_C_*	Bactericidal TB drug rate	0 or 0.5 day^−1^ b
*ε_S_*	Bacteriostatic TB drug efficacy	0 or 0.5^b^
*d*	Drug interaction parameter	0 or 0.75^b^

a M_φ_ denotes “macrophage”.

b 0 for inactive, >0 for active.

For CD4+ T cells the initial and disease-free equilibrium cell count *H_df_* = 1000 mm^−3^ [23]. The proliferation parameter *r_H_* = 0.01 day^−1^ [16], similar to the 0.02 value of Kirschner [40], and the corresponding half-saturation constant *C_H_* = 1000 pathogen mm^−3^ [40]. This represents proliferation of the 1–2% of antigen-specific cells within the total CD4+ population. The upper limit *H_max_* = 1500 mm^−3^ [20]. Natural death rate *μ_HS_* = 0.01 day^−1^ [14], [23]. Consequently the source *s_H_* = *μ_HS_H_df_* = 10 cells mm^−3^ day^−1^.

The eclipse stage of a CD4+ T cell after infection by HIV lasts about 1 day [11]. In the model the loss rates act as soon as a cell enters a new compartment so to counter this effect we set the total eclipse time to about 3 days, with the transition rate to being productively infected *μ_HE_* = 0.25 day^−1^. Hence the sum of half-lives of eclipse and productively infected cells (discussed next) is about 4 days, consistent with the 3–4 days reported for a complete cycle from infection to budding [43]. The HIV-infected CD4+ T cell death rate due to budding of virions is *μ_HV_* = 0.5 day^−1^ (median life-span 1.4 days), consistent with values used in a number of models [11], [13], [19], [22], [23].

CD8+ T cells number about 500 mm^−3^ [52] of which about 2.5–3% are antigen-specific. We set the disease-free resting level *K_df_* = 10 cells mm^−3^, just lower than the 12.5–15 value corresponding to 2.5–3% but somewhat higher than 5 set by Magombedze et al. [42]. These cells proliferate in response to antigen at a rate *r_K_* = 1.5 day^−1^ [11]. They do not increase without bound so following the method of Perelson et al. [20] we set an upper limit at *K_max_* = 100 cells mm^−3^ which is the estimated upper level of 20% of CD8+ cells specific to an antigen. The half-saturation constant (infected cell level) to trigger response is estimated as *C_K_* = 2.5 cells mm^−3^. Althaus and De Boer [11] have a similar Michaelis-Menten constant which depends on cytotoxic efficacy and we have therefore set our value to have efficacy that corresponds to observed low levels of infected cells. Natural death rate is *μ_K_* = 0.5 day^−1^, within the 0.3–1 range used by other models [11], [22] and consequently the source *s_K_* = *μ_K_K* = 5 cells mm^−3^ day^−1^.

The maximum number of cells lysed by a CD8+ cytotoxic T cell is estimated as a maximum of *N_L_* = 50 infected cells per cytotoxic cell per day [11]. This is much higher than values in the range 0.5–3 used in other models [30], [39] and reported by experiment [52] but it is a maximum rate not attained in our simulations so its high value does not play a significant role. Althaus and De Boer [11] present a wide range for the half-saturation constant (1–10^9^ cells mm^−3^) depending on cytotoxic efficacy. We set this as *C_L_* = 2500 infected cells mm^−3^, consistent with 50 cells lysed by the maximum of 100 cytotoxic cells mm^−3^ in the model. In principle, number of cells lysed per day per mm^3^ could be modelled with a Michaelis-Menten saturation function 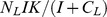 where *I* is the infected cell density. However, in the simulations *I*≪*C_L_* so the approximation 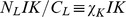 is suitable, where the lysis rate χ*_K_* = *N_L_*/*C_L_* = 0.02 CD8^−1^ day^−1^.

Virion burst size from an infected T cell is *N_H_* = 1000 [16], [42], within the 100–1000 range presented by Kirschner [40] for both T cells and macrophages. This is not a narrowly-determined parameter; values used in recent models range from 100 [22] to 50 000 [19]. The burst size from an HIV-infected macrophage is *N_M_* = 800 [42]. The budding period lasts from about a day for T cells to months for macrophages.

Virion clearance rate due to natural virion decay and neutralising antibodies is *μ_V_* = 4 day^−1^ [22] which is close to values used in other work [14], [39]. This parameter varies in different models, from as low as 1.5 [42] to as high as 23 [11], [23]. We chose the intermediate value which together with other parameter values produces a reasonable pattern of HIV infection. HIV infection rate of T cells is *λ_VH_* = 10^−4^ virion^−1^ day^−1^, close to 9×10^−5^ used by Bauer at al. [39] and in the range they present of 10^−5^–10^−4^. Rate of HIV infection of macrophages is *λ_VM_* = 10^−5^ virion^−1^ day^−1^, set at 10% of the T cell rate [42].

Death rate of CD4+ T cells due to toxic HIV proteins (uninfected bystander death [44], which Alimonti et al. [44] argue is at least as large as death by infection) is estimated as *χ_V_* = 10^−4^ virion^−1^ day^−1^, equal to the HIV infection rate. Our model also includes non-cytoxic action of CD8+ T cells that reduces viral infection and replication [11], [39], [42]. We set *p_I_* = *p_R_* = 0.1(cell/mm^3^)^−1^, similar to those of Magombedze et al. [42] who estimate *p_I_* = 0.075 and *p_R_* = 0.05 for T cells and *p_I_* = 0.85 and *p_R_* = 0.075 for macrophages.

The initial and disease-free level of resting macrophages was set at *M_df_* = 200 cells mm^−3^ [14]. In other models this ranges from 100 [40] to 300 [39], and as high as 500 [35]. Since macrophages are recruited to an infection site in response to pathogen we allow for an increase of the source function by a factor 10, so *r_M_* = 9. The half-saturation recruitment constant was estimated at *C_M_* = 500 bacilli mm^−3^ which is approximately half the maximum level attained in the simulations (higher levels would be associated with large necrotic tubercles and cavities). Natural death rate *μ_MR_* = 0.01 day ^−1^ [35], [42] so *s_M_* = *μ_MR_M_df_* = 2 cells mm^−3^ day ^−1^.

The activation rate of resting macrophages *a_M_* = 0.1 day ^−1^ [39]. The death rate *μ_MA_* = 0.1 day ^−1^, close to 0.07 used by Bauer at al. [39]. Half-saturation constant for activation *C_A_* = 500 bacilli mm^−3^ (Bauer at al. [39] give a wide range of 1–10^4^). Macrophages in the eclipse stage last about a week before dying [28] so the transition rate of *M_E_* to productively MTB-infected is *μ_ME_* = 0.05 day^−1^. Death rate of MTB-infected macrophages (*M_B_*) due to bursting is *μ_MB_* = 0.1 day^−1^ [35], within the range 0.001–0.1 [39]. The death rate of HIV-infected macrophages due to virion budding is *μ_MV_* = 0.01 day^−1^ [35], [42], within the range 0.001–0.1 [39].

MTB burst size *N_B_* = 50 [35], [42] is the number of bacilli released when an MTB-infected macrophage bursts due to a large bacillary load. A macrophage has a carrying capacity of 10–50 bacilli [39] and we assume an average maximum phagocytosis of *N_φ_* = 10 bacilli macrophage^−1^ day^−1^. Following Marino and Kirschner [35] and Magombedze et al. [42] we set the number of bacilli ingested at infection at *N_i_* = *N_B_*/2 = 25, and then the number of bacilli released on lysis of an MTB-infected macrophage is *N_K_* = 35, intermediate between *N_i_* and *N_B_*. This is consistent with the scheme [28] whereby a macrophage becomes necrotic with a load of 32 bacilli. The half-saturation phagocytosis constant is estimated as *C_φ_* = 5000 bacilli mm^−3^. These values are intended as approximate but reasonable indicators of the complex underlying process. The phagocytosis rate *χ_φ_* is calculated from Eq. (25) as 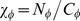 = 0.002 macrophage^−1^ day^−1^. As discussed for lysis, this is an approximation of a Michaelis-Menten function 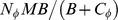 where *B≪C_φ_.* In the simulations, *B* does not exceed 1500 mm^−3^ so it is well below *C_φ_* although it starts to be comparable to *C_φ_*. Making the approximation produces little difference in simulation results and also simplifies analysis of the model. MTB infection rate is calculated from Eq. (17) as 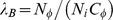 = 8×10^−5^ bacillus^−1^ day^−1^. MTB loss rate we estimate as *μ_B_* = 0.1 day^−1^.

We conclude with miscellaneous parameters. Those for treatment efficacy were set to *ε_P_* = *ε_R_* = 0.75, *ε_S_* = 0.5 and *ε_C_* = 0.5 day^−1^ (compare with e.g. overall *ε_ARV_* = 0.85 [23]) which cleared both infections. The drug interaction parameter *d* = 0 for no interaction and we set *d* = 0.75 for drug interaction. For modelling progression to AIDS, *V_ref_* = 100 virions mm^−3^ to obtain with the other parameters a disease lifetime of about 10 years, and the Weibull cumulative distribution function has scale parameter *σ* = 11.362 years and shape parameter *k* = 2.871 which result in a median of 10 years and a 10%–90% width of 10 years. The viral production increase parameter is set to *p_V_* = 5 in order to produce a maximum 6-fold increase (maximum *R_V_* = 6). These values were selected to produce a pattern consistent with observation. The parameter that determines increase in virion production due to MTB infection was estimated as *p_B_* = 0.2 to produce a significant but not dominant effect, and the scale parameter *B_max_* = 1000 bacilli mm^−3^. Median immune response time *T_IR_* = 14 days. The MTB cure parameter was set arbitrarily to *B_cure_* = 1 bacillus mm^−3^ and undetectable viral load threshold *V_low_* = 0.05 mm^−3^ (50 per milliliter).

## Results

All simulations were run by solving Eq. (9–11, 13, 14, 16, 18–22, 24) numerically with a C++ program. The algorithm is a first-order Euler integrator with time step *Δt* = 1 minute. If a population variable *U_n_* is known at time *t_n_* then after one time-step
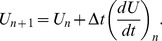
(26)Although this is a low-order method the small time-step keeps the error to a total of 0.24% for an exponential growth rate of 1 day^−1^ that produces a 1000-fold increase over 7 days. This rate is larger than any in the simulations and is approached only by *V* during primary infection.

### Simulation of HIV and MTB infection without treatment

**Figure 2:**
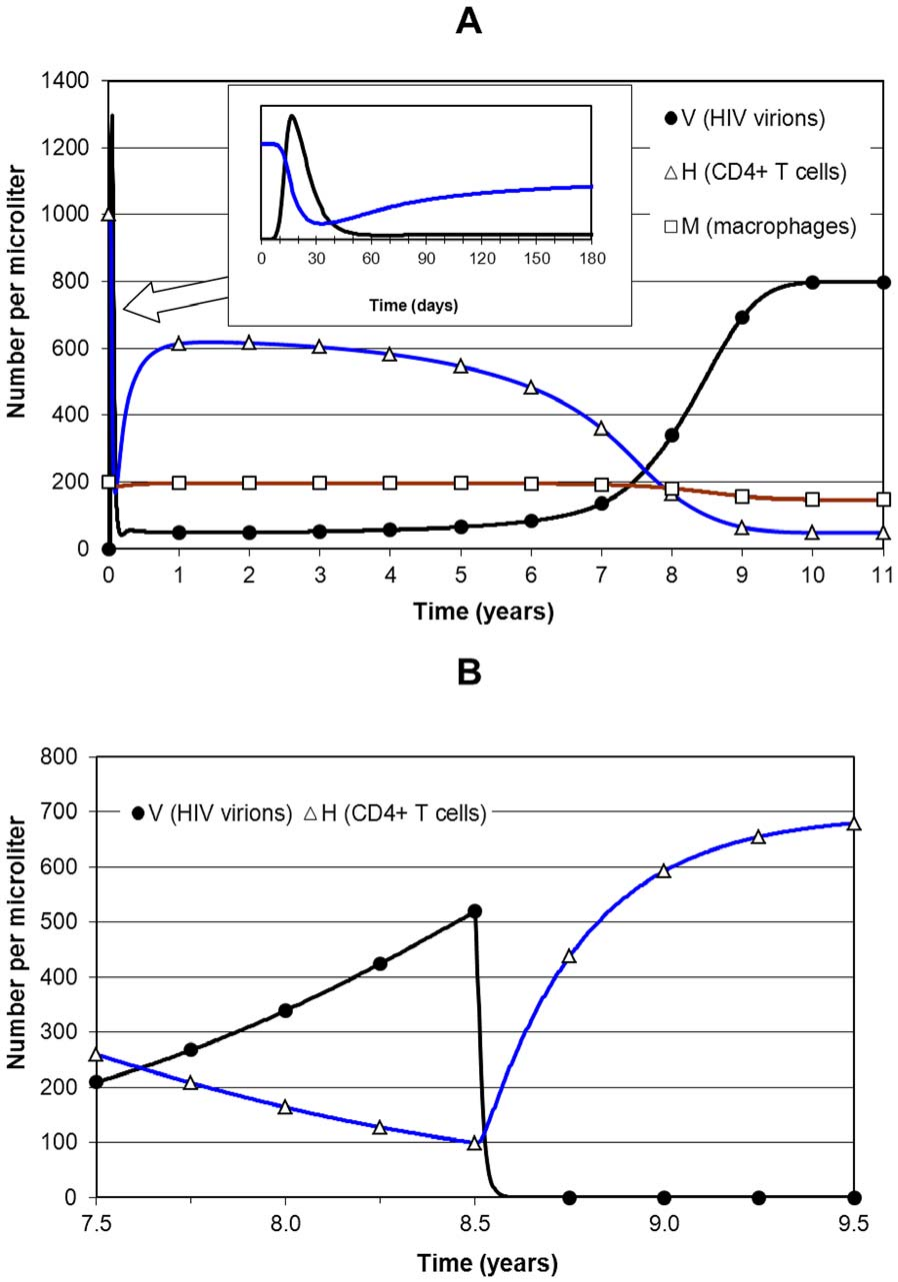
Simulation of HIV infection only and progression to AIDS. Panel A shows an HIV-only simulation over 11 years with the primary stage as an inset. At time *t* = 0 HIV infection starts with viral load *V* = 0.01 µL^−1^, CD4 count = 1000 µL^−1^ and resting macrophage population *M_R_* = 200 µL^−1^. *V* rises to a spike of 1300 within the first month and then settles to a quasi-stable set-point within a year with *V*≈50 and CD4 just above 600. As time advances through the asymptomatic stage (CD4>500) viral replication slowly increases and CD4 source capacity decreases, and this process accelerates as early HIV disease sets in after year 6, progresses to late HIV disease by year 8, and to AIDS by year 10 when CD4 falls below 50 and *V* rises to 800. Panel B shows ART started at 8.5 years when *V* = 520 and CD4 = 99. *V* falls below the undetectable threshold of 0.05 in 7.3 weeks and CD4 recovers over the next 6–12 months to almost 700.

Simulation of HIV infection only is shown in Figure 2A. Time *t* is measured in years, with HIV infection started at *t* = 0 by setting viral load *V* = 0.1 mm^−3^. The model approximates HIV infection dynamics in a manner consistent with clinical observations. Initially (during primary infection, expanded in the inset) there is a short-lived spike in *V* which settles down to a quasi steady-state value (the “set-point”) in the early phase of the asymptomatic stage. It is only quasi steady-state because both viral replication and T cell source capacity are slowly declining (very slowly initially) with integrated viral load. At this stage *V* is about 50 mm^−3^ and CD4+ T cell count is just over 600 mm^−3^. Since all population counts are per mm^3^ (microliter) this unit will be implied in what follows unless stated otherwise. We also refer to CD4+ T cell count simply as “CD4”.

For the first four years CD4 and viral load remain fairly steady and then from years 4–7 CD4 declines gradually to 360 and viral load increases to 135. After the asymptomatic period the integrated viral load has increased and resulted in a declining capacity of the body to generate new CD4+ T cells concurrently with increased viral replication. This simulates immune collapse to AIDS which is firmly established from year 10 when CD4 has fallen below 50 and viral load has risen to almost 800. The macrophage population remains fairly constant at 196 (small reduction due to HIV infection of macrophages) until the AIDS stage when it decreases to about 150.

Figure 2B shows the 2-year period (years 7.5–9.5) with ART started at 8.5 years when *V* = 520 and CD4 = 100. Drug efficacies are *ε_R_ = ε_P_* = 0.75. *V* falls to 0.05 (or 50 per milliliter: undetectable threshold) in 7.3 weeks with over 90% of the decline in 2 weeks. By this time CD4 has recovered to 313 and continues to rise to 680 by 9.5 years and reaches 694 by the start of year 10, 18 months after treatment. CD4 does not return to the pre-HIV level of 1000 because some T cell source capacity has been permanently lost. The 6 months after treatment, during which CD4 recovers to about 600 (85% of the final level), is a time of rapid immune restoration and hence the period of risk of IRIS.

**Figure 3:**
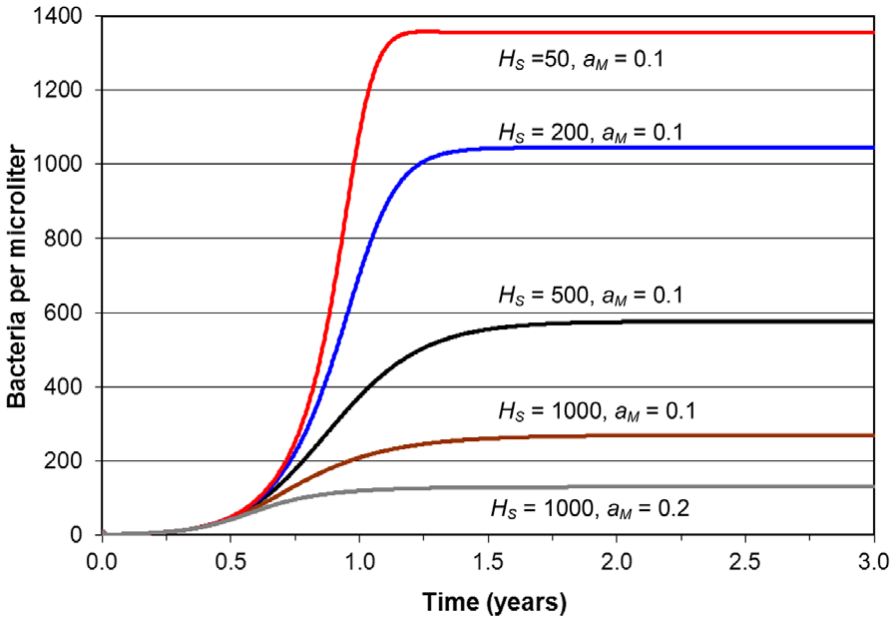
Simulation of MTB infection only. Simulations of MTB infection were run for various fixed CD4+ T cell levels and two macrophage activation rates *a_M_* to show the dependence of infection on immune system strength. MTB infection starts at time *t* = 0 with bacterial level *B* = 10 µL^−1^. CD4 level is kept constant at values as indicated on the curves, for values of *a_M_* as shown. Each curve shows that *B* increases relatively slowly over about 18 months to a maximum level. The maximum increases as immune strength decreases with decreasing values of *a_M_* and CD4.

Figure 3 depicts MTB infection only over 3 years for various parameter values. Infection starts at *t* = 0 with initial bacterial level *B* = 10. After about 18 months *B* reaches a maximum value. Normally the model allows *H_S_* to proliferate in response to *B* but here *H_S_* is kept fixed. The lowest curve has full CD4 capacity (*H_S_* = 1000) and a macrophage activation rate *a_M_* = 0.2 that is double that for all the other curves. Immune suppression is strong and *B* reaches 130. With *a_M_* = 0.1 bacterial level increases to 270. Then as *H_S_* is decreased in steps to 50, the maximum level of *B* continues to rise to 1355. Hence the model simulates active MTB infection that is controlled, but never eliminated, as a function of immune system strength.

**Figure 4:**
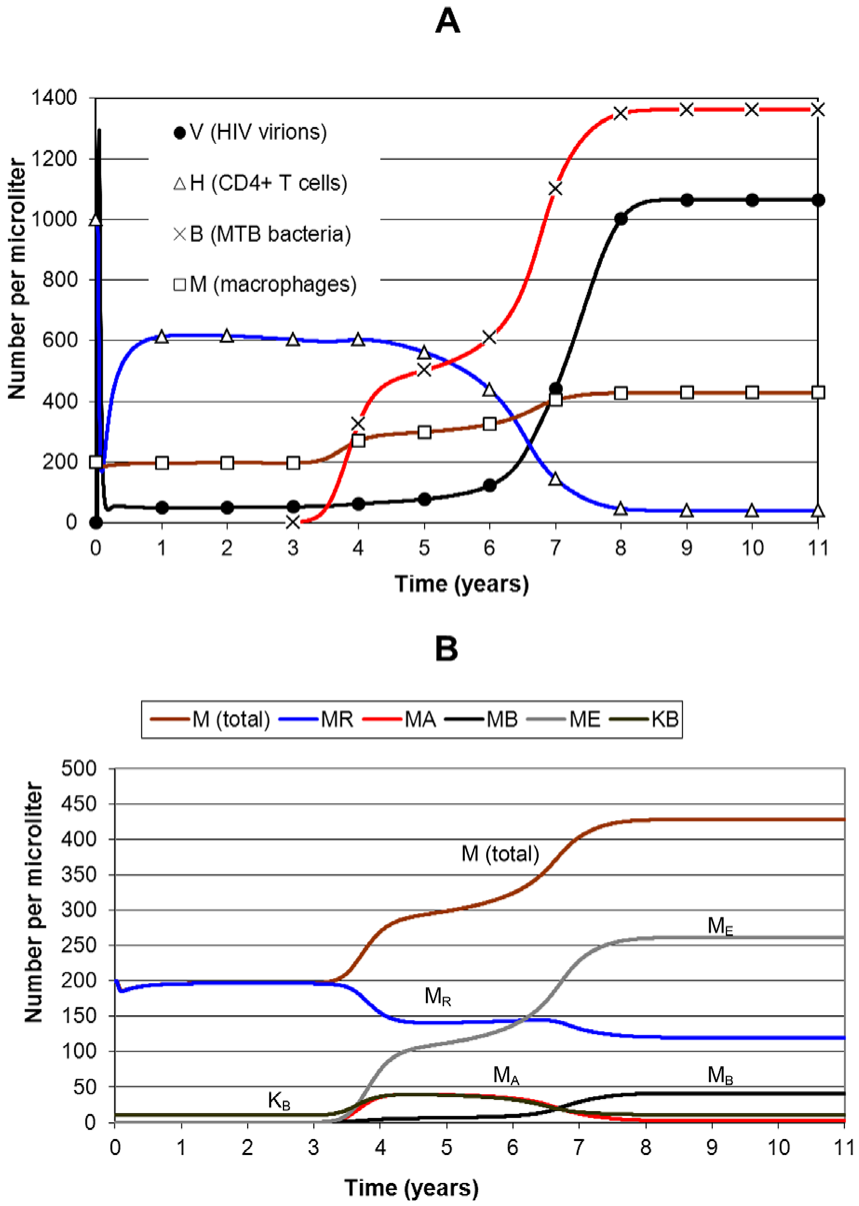
Simulation of HIV-MTB co-infection, without treatment. The panels show different populations in a simulation of HIV-MTB co-infection without treatment. HIV infection starts at *t* = 0 and MTB infection at *t* = 3 years. Panel A shows CD4, viral load, bacterial level and total macrophage population. Since CD4>500 µL^−1^ at *t* = 3 years the immune system is relatively strong and *B* reaches a maximum level that starts to rise again as HIV disease progresses to AIDS. By the AIDS stage, *B* reaches a maximum of 1362. *V* reaches 1065 which is higher than the HIV-only maximum due to increased viral replication induced by MTB. The macrophage population rises from its 200 resting level to about 300 during MTB infection and then to about 430 during AIDS due to a decrease in activation that allows increased MTB infection. Panel B shows the smaller populations. Macrophages are activated by MTB but lost to HIV infection and especially MTB infection, with the loss increasing during AIDS. The CD8+ T cell population *K_B_* proliferates in response to the infections.

HIV-MTB co-infection is modelled in Figure 4. HIV infection starts at *t* = 0 and MTB infection at 3 years. Panel A shows that bacterial level rises over the following year to 400 after which its rise slows due to reasonable immune control. The macrophage population rises due to recruitment stimulated by MTB infection. By year 6 CD4 falls below 500, the immune system starts to collapse, and bacterial level starts to rise and attains a new maximum of 1362. Viral load increases to over 1000, higher than for HIV infection only due to an increase caused by MTB of around 27% in viral replication. Progression to AIDS is also more rapid than for HIV only due to the increased integrated viral burden. CD4 has fallen to below 50 by year 8, almost two years earlier than for HIV alone.

Figure 4B is a plot of the macrophage and CD8+ T cell populations. The productive HIV-infected T cells and macrophages are not shown because their levels are low (less than 10). Resting macrophages (*M_R_*) and MTB-specific CD8+ T cells (*K_B_*) are stable until MTB infection at year 3. After infection the total macrophage population rises to 300 at first and then to 430 during advanced HIV disease. During MTB infection the activated macrophages (*M_A_*) rise rapidly to a level of about 40, somewhat below *M_R_*, and then decline during AIDS because the lack of CD4+ T cells reduces macrophage activation. *K_B_* follows a similar pattern to *M_A_* for the same reasons. The MTB-infected macrophages in the non-productive eclipse stage (*M_E_*) are paralleled at a lower level by the productive population (*M_B_*). Their total (*M_E_+M_B_*) first rises to about 120 (40% MTB infection prevalence rate) and then to 300 (70% prevalence) during AIDS.

### Modelling co-infection treatment strategies

Having tested the model's capacity to simulate HIV-MTB co-infection it is extended to include several combined treatment strategies. Expert reviews [1]–[Bibr pone.0049492-Dean1] [Bibr pone.0049492-Kwara1] [4] recommend starting with TB treatment, with ART delayed according to CD4 count as follows:

- CD4>200: About 6 months (end of TB treatment) because “Drug-drug interactions are better avoided than managed” [1]
- <CD4<200: 2–3 months [1], [4]
- CD4<50: A few weeks [4]

Clinical trials have tested similar timings [6]. SAPIT [5] (for CD4<50, and 50<CD4<200) had delays after start of TB treatment of 15–30 days (earlier ART) and 77–126 days (later ART). The CAMELIA trial [8] (for CD4<200) had early ART at 2 weeks and later ART at 8 weeks.

**Figure 5:**
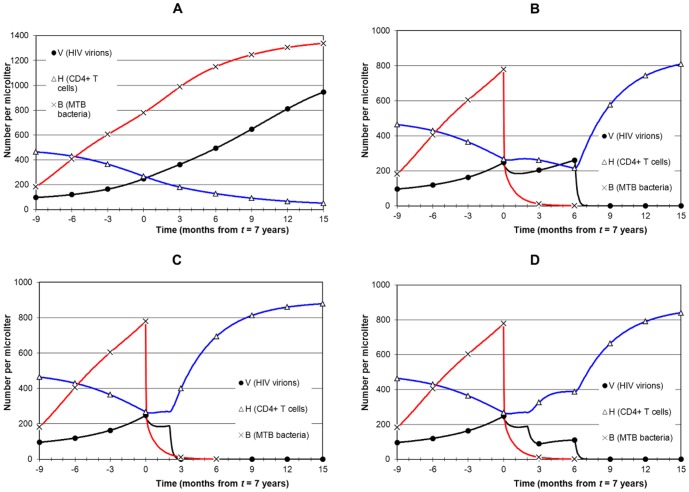
Simulation of combined treatment during early HIV disease. Panel A (no treatment) shows MTB infection that starts at 5years 6 months, and by year 7 when treatment starts bacterial load rises to 779 µL^−1^ and CD4 is 268 µL^−1^. In all cases, TB treatment starts at 7 years and ART after a delay. Panel B shows late ART (strategy 1: delay of 6 months). MTB infection has been eliminated by the time ART begins so there is no drug interaction, no overlap of drug toxicity and no TB IRIS. CD4 falls to about 200 and then recovers. Panel C shows early ART (strategy 2: delay of 2 months) with no drug interaction, which allows CD4 to remain stable and then recover, but there is some overlap of drug toxicities. There is also risk of TB IRIS, but bacterial level is low. Panel D shows the same scenario as for panel C but with drug interaction that moderately increases viral replication and delays CD4 recovery.

**Figure 6:**
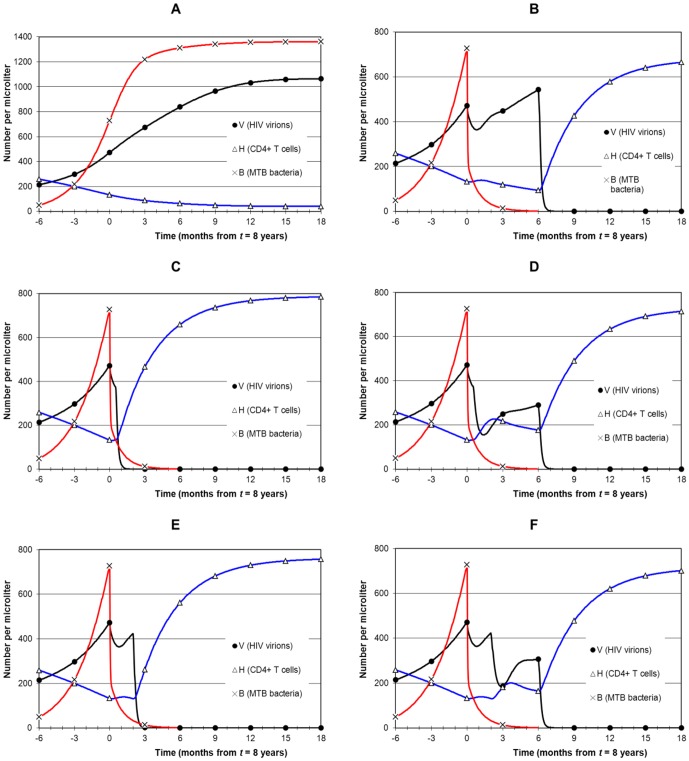
Simulation of combined treatment during late HIV disease. Panel A is the no treatment scenario with MTB infection starting at 7 years. By year 8, *B* = 727 µL^−1^, CD4 = 133 µL^−1^ and *V* = 472 µL^−1^. All treatment scenarios start with TB treatment first at 8 years. Panel B shows ART starting at 8 years 6 months (strategy 1). The outcome is as for Figure 5B except that *V* is much higher and CD4 falls below 100. This puts the patient at higher AIDS-associated risk than with earlier ART. Panel C shows immediate ART starting 15 days after the start of TB treatment (strategy 3), with no drug interaction. CD4 recovery is early but *B* is high during the steepest rise of CD4 so risk of TB IRIS is high. Adding drug interaction (panel D) causes a resurgence of *V* and a delay in CD4 recovery although CD4 remains stable at around 200. Panel E has early ART starting after 2 months with no drug interaction (strategy 2). CD4 remains stable for the 2 month period and then recovers. Bacterial level is relatively low during CD4 recovery so risk of TB IRIS is less than for immediate ART. Panel F adds drug interaction which as in Panel D delays CD4 recovery, but for 6 rather than 2 months.

**Figure 7:**
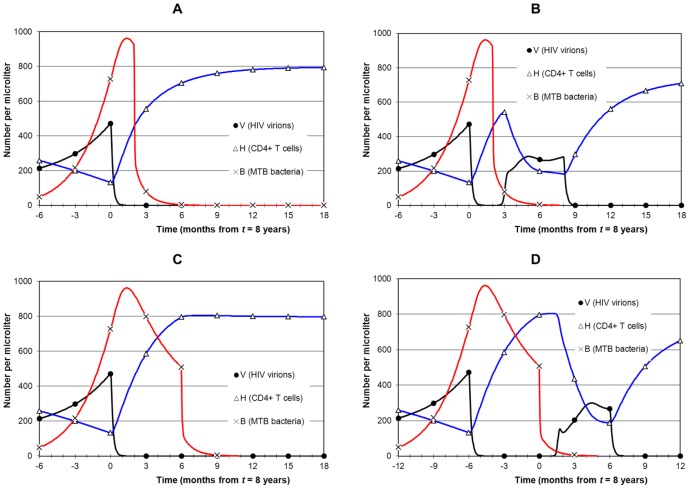
Simulation of ART before TB treatment during late HIV disease. As for Figure 6 this simulation is of late HIV disease but with ART started first at 8 years. In panel A, TB treatment starts 2 months after ART begins (strategy 4). Drug interaction is not included. Viral load falls rapidly and CD4 recovers immediately. The strengthened immune system slows and then reverses bacterial growth just before TB treatment starts, but MTB infection remains strong. A high risk of TB IRIS as well as ongoing TB are negative outcomes. Drug interaction (panel B) worsens the situation with a resurgence of *V* and a delay in CD4 recovery. Delaying ART by 6 months to allow the immune system a longer time to act against TB (strategy 5: panel C) simply extends the period of high bacterial load. Drug interaction (panel D) allows resurgence of *V* and a renewed collapse of CD4 before it recovers again.

**Figure 8:**
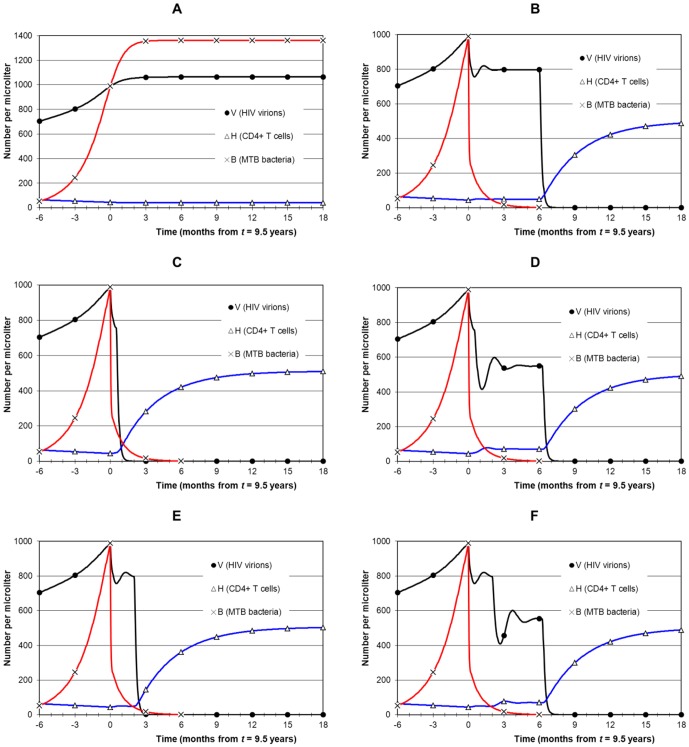
Simulation of combined treatment during AIDS. Panel A shows the no-treatment AIDS scenario. At 9.5 years *V* = 989, CD4 = 44 and *B* = 989. TB treatment starts first in all strategies. Panel B shows ART after 6 months (strategy 1). AIDS-associated risk is high during the prolonged period of high *V* and low CD4. Panel C applies immediate ART after 15 days, with no drug interaction (strategy 3). CD4 recovery is immediate although risk of TB IRIS is high due to relatively high bacterial level. Adding drug interaction (panel D) negates the benefits of immediate ART because *V* remains high and CD4 remains low. A 2 month delay of ART (strategy 2: panel E) has a similar outcome as in panel C. Again, drug interaction (panel F) negates the benefits of early ART and prolongs high *V* and low CD4.

To cover the reviewed range of timings we tested treatment strategies with delays of 15 days, 2 months and 6 months between start of TB treatment and start of ART. In addition, we tested ART first. We considered three timings for TB presentation of an HIV-infected individual:

1. Early HIV disease, when CD4 is between 200 and 350. MTB infection starts at 5.5 years and earliest treatment at 7 years (Figure 5).
2. Late HIV disease, when CD4 is between 50 and 200. MTB infection starts at 7 years and earliest treatment at 8 years (Figures 6 and 7).
3. AIDS stage, when CD4 is below 50. MTB infection starts at 8.5 years and earliest treatment at 9.5 years (Figure 8).

The MTB infection may be new, or reactivation of a latent infection, and in either case we assume that the infection becomes active fairly rapidly due to a weakened immune system. We assume further that after the bacterial load has exceeded about 500 the individual presents with symptoms that lead to treatment a short time later, and that bacterial load significantly higher than 500 could be fatal if it continues for more than a few months. TB treatment is administered for 6 months. For each timing one or more of the following strategies are implemented:

1. TB treatment first, followed after 6 months by ART
2. TB treatment first, followed after 2 months by ART
3. TB treatment first, followed after 15 days by ART
4. ART first, followed after 2 months by TB treatment
5. ART first, followed after 6 months by TB treatment

We set ARV parameters *ε_R_* = *ε_P_* = 0.75 and anti-TB drug parameters *ε_C_* = *ε_S_* = 0.5. These values clear the infections in reasonable time (*B* below 1 in 6 months, *V* below 0.05 in 8 weeks). In strategies 2–5 we assume that drug interaction may be operating in which case we set the efficacy reduction parameter *d* to 0.75. Strategies 4 and 5 are not recommended by expert reviews [1]–[Bibr pone.0049492-Dean1] [Bibr pone.0049492-Kwara1] [4] but are included for completeness in order to see what happens (according to the model) if ART starts first. The idea is that a recovered (or recovering) immune system could assist TB treatment in recovery from MTB infection. Key values for the timings and strategies are summarised in Table 3.

**Table 3:** Summary of HIV-MTB co-infection and treatment scenarios.

Infection or treatment scenario	Time	CD4	HIV	MTB
	(years)	(mm^−3^)	(mm^−3^)	(mm^−3^)
**HIV-MTB co-infection, no treatment**				
HIV infection starts	0	1000	0.01	0
Early asymptomatic stage	1.5	619	49	0
MTB infection starts	3	604	52	10
Late asymptomatic stage	6	439	122	609
AIDS	9	40	1065	1362
**Early HIV disease (200<CD4<350)**				
TB treatment starts first	7	268	248	779
ART starts 2 months later	7.17	269	189	29
ART starts 6 months later	7.5	214	261	0
**Late HIV disease (50<CD4<200)**				
TB treatment starts first	8	133	472	727
ART starts 15 days later	8.04	126	375	134
ART starts 2 months later	8.17	131	423	33
ART starts 6 months later	8.5	93	543	0
**Late HIV disease (50<CD4<200)**				
ART starts first	8	133	472	727
TB treatment starts 2 months later	8.17	455	0.01	923
TB treatment starts 6 months later	8.5	796	3×10^−8^	508
**AIDS (CD4<50)**				
TB treatment starts first	9.5	44	989	989
ART starts 15 days later	9.54	49	757	179
ART starts 2 months later	9.67	49	796	44
ART starts 6 months later	10	49	799	6

### Timing 1: Early HIV disease

Figure 5 shows presentation of TB during early HIV disease. MTB infection starts at 5 years 6 months. Panel A shows the scenario without treatment. By year 7, *B* has increased to 779 and *V* to 248, and CD4 has fallen to 268. Panel B shows strategy 1: TB treatment first at 7 years, followed by ART 6 months later on cessation of TB treatment. *B* falls rapidly during the first 3 months of treatment and is eliminated (*B*<1) in 25 weeks. Then ART reduces HIV below the undetectable threshold (*V*<0.05) in 7 weeks. Immune reconstitution follows with a rapid rise in CD4 over 6 months.

Figure 5C shows strategy 2: ART started after 2 months of TB treatment, with no drug interaction. Reduction in *B* has the positive secondary effect of reducing *V*. There is earlier immune reconstitution than for strategy 1, with risk of TB IRIS only during the late phase of TB treatment when *B* is low and the patient is recovering from TB. However, drug interaction (Figure 5D) results in diminished suppression of viral load after ART starts (compared with Figure 5C) and hence a delayed recovery of CD4. There is not much benefit from this strategy compared to starting ART 6 months after TB treatment, and the risk of TB IRIS is higher. However, this is all for early HIV disease; the differences between strategies is more marked for the late HIV disease timing.

### Timing 2: Late HIV disease

Figure 6 shows TB presentation during late HIV disease. MTB infection starts at 7 years, and by 8 years *B* = 727, *V* = 472 and CD4 = 133. The no-treatment scenario is presented in Figure 6A. ART 6 months after TB treatment starts (strategy 1) is shown in Figure 6B. *V* dips after TB treatment but remains high (above 350). CD4 remains low (after a minor initial increase) and falls slowly to below 100 by the start of ART. Once ART is initiated CD4 recovers. Figure 6C displays strategy 3: ART starts 15 days after TB treatment, with no drug interaction. Both infections decline rapidly, which is the best outcome, but the rapid rise in CD4 overlaps with the early phase of treatment when the patient still has TB. With drug interaction (Figure 6D) the outcome is not so positive: *V* rebounds and recovery of CD4 is delayed by 6 months. Finally, strategy 2 (ART 2 months after TB treatment) is shown in Figure 6E, with no drug interaction. Since CD4 recovery occurs when *B* is low there is lower risk of TB IRIS than for earlier ART. The negative aspect is a 2 month extension of low CD4. Figure 6F adds drug interaction, which delays CD4 recovery in a similar manner to that shown in Figure 6D but is still better than a 6 month delay in ART if compared with Figure 6B.

Although starting ART first is not a recommended strategy [1]–[4], we investigate it for completeness in Figure 7 with the motivation that once there is sufficient immune reconstitution after ART it could help TB treatment. Figure 7A shows strategy 4: TB treatment starts 2 months after ART, with no drug interaction. Due to suppression of viral load and recovery of CD4 the rise in *B* is reversed but *B* remains high so the risk of death from TB remains present. The patient has active TB that continues for 2 months at a high level and overlaps with CD4 recovery so risk of TB IRIS is high. The situation is aggravated by drug interaction (Figure 7B) by a resurgence of *V* when the anti-TB drugs reduce ARV drug absorption. There is an associated fall in CD4 and then the patient experiences immune reconstitution a second time. Strategy 5 (Figure 7C: TB treatment starts 6 months after ART) extends active TB by 6 months (with, however, some further reduction of *B* due to improved immune response) with greater overlap of potential IRIS and TB. Adding drug interaction (Figure 7D) aggravates the situation with a similar pattern as in Figure 7B.

### Timing 3: AIDS

The final timing is presentation of TB during AIDS, when CD4 is below 50 (Figure 8). TB treatment had to be extended by one week (to 6.25 months) in order to clear MTB infection. Panel A shows the no-treatment scenario. After MTB infection at 8 years 6 months, *B* rises to about 1000 by 9 years 6 months, when *V* is also about 1000. CD4 is below 50, and remains stable in the model although in reality it could decline. For the purpose of illustration, Figure 8B shows TB treatment first at 9 years 6 months followed by ART 6 months later (strategy 1). Although TB IRIS and drug interaction are avoided, and MTB is eliminated, the continued high viral load and low CD4 over 6 months pose significant risk of AIDS death. Strategy 3 with no drug interaction (Figure 8C) suppresses both infections rapidly and allows CD4 to recover immediately, although with a risk of TB IRIS. Drug interaction (Figure 8D) extends high *V* (although at 550 rather than 1000) and low CD4 (slightly improved to 70) for 6 months. Strategy 2 is shown in Figure 8E (no drug interaction). Risk of TB IRIS is diminished at the cost of a 2 month extension of low CD4. With drug interaction (Figure 8F) the situation is similar to Figure 8D.

## Discussion

It is of vital importance that we understand treatment of HIV-MTB co-infection, which can be helped through interdisciplinary collaboration. We have presented a new model which is able to simulate HIV-MTB co-infection and test various combined treatment strategies. The model of Magombedze et al. [42] is similar, and an important basis from which ours was developed, but there are several differences. Their model has one population of uninfected macrophages whereas ours has separate resting and activated populations. Our model simulates HIV disease progression whereas theirs does not. Our model includes 2 ARVs and 2 anti-TB drugs whereas they have 3 of each. Finally, they tested simultaneous treatment or one treatment only whereas we tested combined treatment with different timings for each therapy. Our model has most similarity to that of Magombedze et al. [42] in that it has intermediate complexity: cytokines are not included but it does include separate CD4+ and CD8+ T cell populations as well as resting and activated macrophages.

Various treatment strategies have been tested but we have not yet weighed the results. It is beyond the scope of this study to estimate probabilities of outcomes, so to decide between strategies we propose a simple method based on a set of risks, assuming that the risk of drug interaction is avoided by a suitable choice of drugs. The risks have quantitative measures to classify them qualitatively as low, medium or high but we do not attach a probability to each risk level. The risks are listed below, with definitions given for medium and high risk, and low risk assumed otherwise.

1. AIDS-related death due to low CD4 count. Risk is medium if CD4<50 for 2 months, or 50<CD4<200 for 6 months. Risk is high if CD4<50 for 6 months
2. TB-related death due to high MTB load (*B*>500). Risk is medium if high MTB load persists for 2 months, and high if it persists for 6 months
3. Adverse effects of overlap of drug toxicities, measured by timing and duration of overlap. Risk is high during the first 2 month phase of TB treatment, and medium during the second 4 month phase
4. TB IRIS, measured by MTB load during the immune reconstitution period (6 months of rapid increase of CD4). Risk is high if *B*>50, and medium if 1<*B*<50.

Based on these risks, strategies are chosen by the following criteria, in order of preference:

1. Low risks only
2. Low risk of both AIDS-related and TB-related death
3. Low and medium risks only

Table 4 shows risk levels for the simulated treatment strategies. In general, early ART after TB treatment is better than late ART provided that TB IRIS and drug interaction are minimised. This depends on HIV disease stage. For early HIV disease a longer delay of ART is acceptable because CD4 remains relatively high. Strategy 1 is best since it has low risks only whereas strategy 2 (earlier ART) has medium levels of risks 3 and 4. For late HIV disease strategy 2 is best: initiate ART 2 months after TB treatment starts. A 6 month delay has risk of AIDS death at medium level whereas a 15 day delay has high levels of risks 3 and 4. Strategies 4 and 5 (ART first) for late HIV disease are inferior to strategies 2 and 3 because of medium to high risk of TB-related death and high levels of risks 3 and 4. During AIDS, strategy 3 is best (15 day delay of ART) because risk of AIDS-related death is low compared to strategies 1 and 2. Strategy 3 minimises the time spent with critically low CD4. Medium risk of AIDS-related death in strategy 2 is a worse outcome than high levels of risks 3 and 4 in strategy 3. Drug interaction complicates the strategies, especially during AIDS, when resurgence of viral load and associated suppression of CD4 increase the risks of AIDS-related death and TB IRIS. These results are consistent with expert recommendations [1]–[Bibr pone.0049492-Dean1] [Bibr pone.0049492-Kwara1] [4] and with results from clinical trials (SAPIT [5], [7], CAMELIA [8], and [9]).

**Table 4:** Summary of treatment outcomes.

	Risks^a^			
	1	2	3	4
Treatment strategy	AIDS death	TB death	Drug overlap	TB IRIS
**Early HIV disease, TB treatment first**				
ART starts 6 months later (strategy 1)	low	low	low	low
ART starts 2 months later (strategy 2)	low	low	medium	medium
**Late HIV disease, TB treatment first**				
ART starts 6 months later (strategy 1)	medium	low	low	low
ART starts 2 months later (strategy 2)	low	low	medium	medium
ART starts 15 days later (strategy 3)	low	low	high	high
**Late HIV disease, ART first**				
TB treatment starts 2 months later (strategy 4)	low	medium	high	high
TB treatment starts 6 months later (strategy 5)	low	high	high	high
**AIDS, TB treatment first**				
ART starts 6 months later (strategy 1)	high	low	low	low
ART starts 2 months later (strategy 2)	medium	low	medium	medium
ART starts 15 days later (strategy 3)	low	low	high	high

a The four risks are negative events that should be avoided. Risk is low, medium or high. A strategy is preferred first if it has low risks only, second if it has low risk of both AIDS-related and TB-related death, and third if it has low and medium risks only.

What new knowledge do the simulations provide? First, the model agrees with expert reviews and clinical results and supports current understanding. If the simulations were to diverge from observations it would be necessary to review the mechanisms in the model. But in fact there is convergence, which is evidence that the theory in the model is consistent with empirical data. Second, the model was able to test, however approximately, a strategy to treat HIV first. This strategy is neither proposed in expert reviews [1]–[Bibr pone.0049492-Dean1] [Bibr pone.0049492-Kwara1] [4] nor included in clinical trials [5]–[Bibr pone.0049492-Piggott1] [Bibr pone.0049492-AbdoolKarim2] [Bibr pone.0049492-Blanc1] [9] but is nevertheless a strategy that is possible and that can easily be tested with a computer model. The results indicate that it is not a good strategy. The third aspect highlighted by the simulations is the potential risk of drug interaction. In all cases the benefits of early ART were negated by drug interaction that increased viral load and suppressed CD4 count. Especially in resource-poor settings the risk of drug interaction cannot be ignored.

In summary, the model is able to simulate the disease-treatment process, albeit in a highly-simplified and approximate manner. It is far from capturing the full complexity of the system, but it is nevertheless based on biomedical science and clinical data. Its features could be improved to provide an increasingly realistic simulation that serves as a testing and decision tool. The criteria for deciding between strategies could be coded into a more robust quantitative measure. This could incorporate rate of change of CD4 as a measure of IRIS risk, as well as viral and bacterial load, drug toxicity, and improved measures of drug interaction. Such a model could help in designing new clinical trials, with a range of scenarios tested in advance.

Further work could address mechanisms of HIV disease progression, suppressed, latent and active MTB infection, and granuloma and tubercle formation. TB drug resistance could be modelled. As a step toward multi-scale simulation the host-pathogen model could be embedded in a population-level epidemiological model. All of these avenues will be navigated best by multi-disciplinary collaboration.

## Supporting Information

Appendix S1 **Analysis of the model.** The analysis has three parts. First, the quasi-equilibrium state is analysed on time-scales short compared with the time scale of progression to AIDS. Second, simulations are run for a reduced model that does not have activated macrophages *M_A_* or the eclipse-stage populations *H_E_* and *M_E_*, in order to investigate the necessity of these populations. Third, simulations are run without the immune response to see to what extent it is necessary for modelling treatment of HIV-MTB co-infection and treatment.
(PDF)

Figure S1 **CD8+ killer T cell dependence on infected cell level.** CD8+ killer T cell level *K* in the equilibrium state is plotted against infected cell level *I* for various values of *h = H_S_/H_df_*. *K* is the solution of the quadratic equation S2. The *K* curves all lie between *K_df_* = 10 and *K_max_* = 100 cells per microliter. Each curve rises with increasing *I* as proliferation of *K* is stimulated, and *K* stabilises above the *I* half-saturation level of 2.5. The maximum of *K* decreases as *h* decreases.
(PDF)

Figure S2 **CD4+ helper T cell dependence on pathogen level.** CD4+ helper T cell level *H_S_* in the equilibrium state is plotted against the combined pathogen level *P* = *V* (HIV)+*B* (MTB). Various curves are shown for different values of HIV-specific CD8+ killer T cell level *K_V_* and HIV proportion *V*/*P*. The upper curve (*V*/*P* = 0, *K_V_* = 50) is almost constant since there is no loss of *H_S_* to infection and rises slightly with *B* due to increased proliferation. The curves with *V*/*P*>0 decrease with pathogen level even for a small proportion of *V*. Three curves are shown keeping *V*/*P* constant but varying *K_V_* from *K_df_* to *K_max_*. Although there is some dependence on *K_V_* (*H_S_* decreases as *K_V_* decreases) the main dependence is on *V*, by loss to HIV infection.
(PDF)

Figure S3 **Macrophage populations as functions of MTB level.** Equilibrium macrophage levels are plotted against bacterial load *B*. The dashed line is the total of macrophages in the following states: resting *M_R_*, activated *M_A_*, MTB-infected eclipse-stage *M_E_* and productively infected *M_B_*. Total macrophage population increases with *B* and stabilises above the half-saturation level of *B* = 500. Resting macrophages rise at first from the disease-free level of 200 but then fall due to loss to infection. Activated macrophages increase and then stabilise above the half-saturation level, also *B* = 500, but fall slightly at high *B*. Eclipse-stage and productive-stage populations both rise with *B*.
(PDF)

Figure S4 **Dependence of MTB load on CD4+ and CD8+ T cell levels.** Equilibrium bacterial load *B* is plotted against MTB-specific CD8+ killer T cell level *K_B_* in panel A and against *h* = *H_S_*/*H_df_* in panel B. Curves are plotted for various values of resting macrophage population *M_R_* in panel A and of activation parameter *a_M_* in panel B. Panel A shows that *B* depends strongly on *K_B_* in the range 0–20 cells per microliter but does not vary much as *K_B_* increases to 100. There is less dependence on *M_R_*. Panel B shows that *B* decreases strongly with *h* = *H_S_*/*H_df_* and with increasing *a_M_*. This is due to the dependence of the activated macrophage population on the product *ha_M_*.
(PDF)

Figure S5 **Simulation of HIV-MTB co-infection with reduced model.** The graph shows the same scenario as in Figures 2A and 4A, but with the reduced model in which *M_A_, M_E_*, and *H_E_* are omitted. The absence of eclipse-stage populations results in shortened time-lags. Panel A shows the HIV-only simulation in which the initial spike in *V* is much sooner (day 4) and much higher (30 000 mm^−3^) than for the full model. Panel B (HIV-MTB co-infection) shows that MTB infection results in a sudden rise of bacterial load to its maximum level, which does not model the characteristic slow growth of MTB.
(PDF)

Figure S6 **Simulation with full model but without immune response.** To show dependence of the system on immune response, the full model is run as for Figures 2A and 4A but without macrophage activation or lysis by CD8+ killer T cells. However, the weak response of ingestion of MTB by resting macrophages is retained otherwise there would be no bacterial growth. Results without immune response are shown by solid lines and with immune response by markers. The simulation is compared in what follows to the full model. Panel A (HIV-only) shows that the initial spike in *V* is sooner and much higher. Due to initially high viral load, *H_S_* falls more at first and consequently *V* is lower later on since it has fewer target cells to infect, so *H_S_* has a higher set-point level. Transition to AIDS is short (6 months) and abrupt. Panel B shows HIV-MTB co-infection. MTB infection leads to a rapid rise in *B* (since there are neither activated macrophages nor CD8+ T cells to control infection) which triggers an immediate transition to AIDS with much higher viral and bacterial loads than in the model with the immune response.
(PDF)
