# Supplementary material for: Modelling HIV and MTB Co-Infection Including Combined Treatment Strategies
Source: PLoS One. 2012 Nov 28;7(11):e49492. doi: 10.1371/journal.pone.0049492 (PMC3509125; doi:10.1371/journal.pone.0049492)
Supplement: Appendix S1 — Analysis of the model. The analysis has three parts. First, the quasi-equilibrium state is analysed on time-scales short compared with the time scale of progression to AIDS. Second, simulations are run for a reduced model that does not have activated macrophages MA or the eclipse-stage populations HE and ME, in order to investigate the necessity of these populations. Third, simulations are run without the immune response to see to what extent it is necessary for modelling treatment of HIV-MTB co-infection and treatment. (PDF) [file pone.0049492.s001.pdf]

Appendix S1. Analysis of the Model

The model has 60 parameters, listed in Table 2, not counting initial B and V values in Table 1. B_{cure} is arbitrary but small and V_{low} is a definition. A number of parameters are not independent but are used to derive other parameters. Hence the model depends on 46 independent parameters, of which 5 are for drugs and 41 govern the biological system: 3 for HIV disease progression, 12 for immune response, 12 for HIV and MTB, and 14 for homeostatic balance of the 10 immune system populations.

The model has no strict equilibrium state due to HIV disease progression mediated by the function Q. However, we consider here the slowly-shifting quasi-equilibrium (without treatment) on a time-scale short compared to the time-scale on which Q varies. It is found by setting the time-evolution equations to zero and solving for the population variables, with Q assumed to be constant and $\rho_{B,V} = 1$. The disease-free equilibrium has $H_S = H_{df}$, $M_R = M_{df}$, $K_V = K_B = K_{df}$ and the other 8 populations at zero. In the presence of pathogen, we first find the equilibrium states of H_S, K_V and K_B. The K cell equations (13, 14) have the form

$$\frac{dK}{dt} = s_K - \mu_K K + \pi_K K\left(1 - \frac{K}{K_{\max}}\right) \tag{S1}$$

where the proliferation factor $\pi_K \equiv hr_K\, I/(I + C_K)$ and I is the infected cell population. Equilibrium only exists for $K_{df} < K < K_{max}$ and is the positive solution of

$$\frac{\pi_K}{K_{\max}} K^2 + (\mu_K - \pi_K)K - s_K = 0\,. \tag{S2}$$

Figure S1 shows the equilibrium value of K plotted against I for several values of h. The curves lie between K_{df} and K_{max} and decline with h.

H_S has similar behaviour to K except that its proliferation term does not depend on h, and it has loss terms due to V. It has no equilibrium value above H_{max} but in the presence of pathogen it does have equilibrium values below H_{df}. As for K we define $\pi_H \equiv r_H\,(V + B)/(V + B + C_H)$, and set Eq. (9) to zero, rearrange and then solve

$$\frac{\pi_H}{H_{\max}} H_S^2 + \left[(f_I \lambda_{VH} + \chi_V)V + \mu_{HS} - \pi_H\right]H_S - R_C s_H = 0\,. \tag{S3}$$

Figure S2 shows equlibria of H_S as a function of total pathogen $P = V+B$ for various values of K and V/P. The graph shows that H_S depends mainly on V.

The resting macrophage population has equilibrium

$$M_R = \frac{R_{MR}}{c_{losses}}$$

(S4)

where R_{MR} is given by Eq. (15) and c_{losses} denotes the sum of coefficients of M_R associated with losses:

$$c_{losses} = ha_M \frac{B}{B+C_A} + \mu_{MR} + f_I \lambda_{VM} V + \lambda_B B \,.$$

(S5)

The activated macrophages, eclipse-stage and infected populations have equilibria which are straightforward to derive from Eq. (10, 11, 18-21, S4). First we define the activation factor α as

$$\alpha \equiv \left(\frac{h\, a_M}{\mu_{MA} + f_I \lambda_{VM} V} \right) \left(\frac{B}{B+C_A} \right).$$

(S6)

Then the equilibria are:

$$M_A = \alpha M_R$$

(S7)

$$H_E = \frac{f_I \lambda_{VH} V}{\mu_{HE}} H_S$$

(S8)

$$M_E = \frac{\lambda_B B}{\mu_{ME}} M_R$$

(S9)

$$H_V = \frac{f_I \lambda_{VH} V}{\mu_{HV} + \chi_K K_V} H_S$$

(S10)

$$M_V = \frac{f_I \lambda_{VM} V(1+\alpha)}{\mu_{MV} + \chi_K K_V} M_R$$

(S11)

$$M_B = \frac{\lambda_B B}{\mu_{MB} + \chi_K K_B} M_R$$

(S12)

The equilibrium values for the macrophage populations are plotted in Figure S3 as a function of *B*.

Now we consider the pathogens. HIV has equilibrium

$$V = \frac{R_V R_B f_R}{\mu_V}\left(N_H \mu_{HV} H_V + N_M \mu_{MV} M_V\right)$$

(S13)

which on substitution for H_V and M_V from Eq. S10 and S11 yields the relation

$$\frac{N_H \lambda_{VH} H_S}{1+\chi_K K_V/\mu_{HV}} + \frac{N_M \lambda_{VM}(1+\alpha) M_R}{1+\chi_K K_V/\mu_{MV}} = \frac{\mu_V}{R_V R_B f_R f_I}.$$

(S14)

Let $f_{KH} \equiv (1+\chi_K K_V/\mu_{HV})^{-1}$. Then if the macrophage-dependent term is neglected (since it is much smaller than the T cell term) the H_S equilibrium is approximately

$$H_S \approx \frac{\mu_V}{f_{KH} f_R f_I R_V R_B N_H \lambda_{VH}}.$$

(S15)

MTB grows only by intracellular replication so the primary weak immune response (ingestion by resting macrophages) is essential in the model for bacterial growth. In equilibrium, Eq. (24) for time-evolution of *B* is set to zero and after substitution for M_B from Eq. (S12) and χ_ϕ from Eq. (25) it may be rewritten as

$$\frac{dB}{dt} = \lambda_B M_R B\left(N_R - \alpha N_i\right) - \mu_B \frac{B^2}{B_{\max}} = 0$$

(S16)

where N_R is nett bacterial release per macrophage infection, given by

$$N_R = \frac{\mu_{MB}(N_B - N_i) + \chi_K K_B (N_K - N_i)}{\mu_{MB} + \chi_K K_B}.$$

(S17)

In this model, N_R is positive since $N_i < N_K < N_B$. If there is no activation ($\alpha = 0$) in Eq. (S16) then $B = B_{\max}\lambda_B N_R M_R / \mu_B$. If $\mu_B = 0$ then in equilibrium

$$B = \frac{C_A(N_R/N_i)}{\alpha_{\max} - (N_R/N_i)}$$

(S18)

where α_{max} is the limit of α for $B >> C_A$. Thus in the absence of the logistic limit intracellular growth is controlled by activation provided that $\alpha_{max} > N_R/N_i$. Lysis reduces but does not stop intracellular growth because $N_R > 0$ for all K_B. If activation is not high enough then bacterial growth is only limited by the logistic loss term. In this case the equilibrium value of B is found by solving the following quadratic equation derived from Eq. (S16):

$$\frac{\mu_B}{B_{\max}}B^2 + \left[\frac{\mu_B}{B_{\max}}C_A + \lambda_B M_R\left(\frac{ha_M}{\mu_{MA} + f_I\lambda_{VM}V}N_i - N_R\right)\right]B - \lambda_B M_R N_R C_A = 0 \ .$$

(S19)

Figure S4 shows, for $V = 0$, how B varies with K_B for various values of M_R and how B varies with h for various values of a_M.

We now consider a reduced model for comparison with the full model, to see whether a somewhat simpler model may be used. The reduced model omits H_E, M_E and M_A and is obtained from the full model by substituting from Eq. (S7-S9) into Eq. (11, 19, 21, 24). This is equivalent to instant macrophage activation and short life-spans for eclipse populations. A simulation with the reduced model is shown and compared with the full model in Figure S5. Panel A (HIV-only) shows that the transition to AIDS is acceptably represented, although the peak in V during primary infection is earlier (day 4) and much higher (about 30 000 mm^{-3}). However, panel B (HIV-MTB co-infection) shows that MTB infection is much more rapid. The final equilibria of the models are the same, as expected. Although the reduced and full models produce similar results in the sense that there is a transition to AIDS, the full model is preferred because it includes biologically realistic time-lags. Since the model's primary application is to timing of treatment, the preferred model is that which better simulates timing of the biological processes.

Finally we present the full model without immune response to investigate whether the immune response is really necessary in the model in order to simulate

treatment. The lysis rate χ_K and activation parameter a_M are set to zero but the initial response of ingestion of MTB by resting macrophages is retained or else there would be no bacterial growth. Figure S6A shows that the initial spike in V is sooner and higher than with immune response, and transition to AIDS occurs in 6 months with an abrupt fall in H_S. Figure S6B shows that MTB infection results in a sudden rise in B which triggers immediate transition to AIDS. These results show that removing the immune response produces rapid changes in the system that are not suitable for modelling treatment timing strategies.
