# Supplementary figures and images for: Modelling HIV and MTB Co-Infection Including Combined Treatment Strategies
Source: PLoS One. 2012 Nov 28;7(11):e49492. doi: 10.1371/journal.pone.0049492 (PMC3509125; doi:10.1371/journal.pone.0049492)

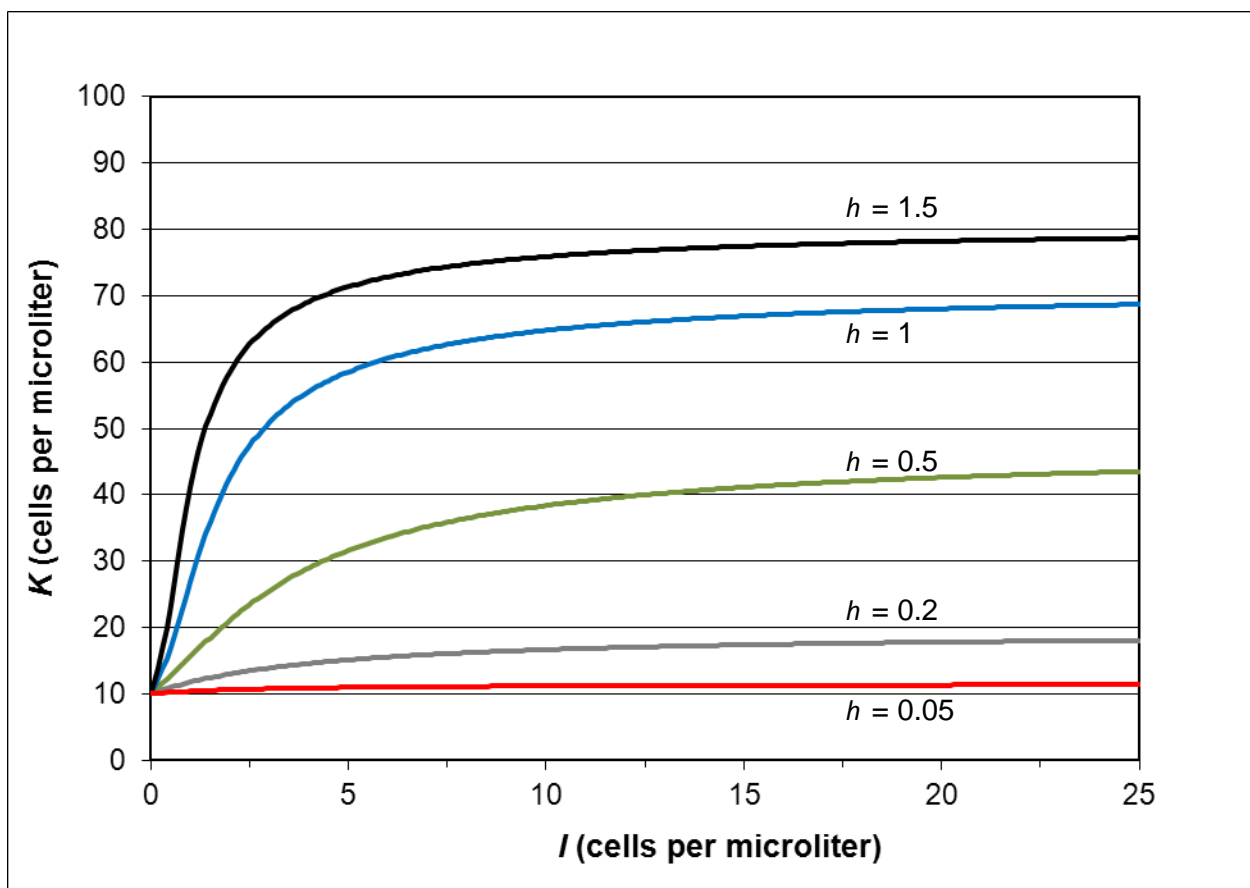

Supplement: Figure S1 — CD8+ killer T cell dependence on infected cell level. CD8+ killer T cell level K in the equilibrium state is plotted against infected cell level I for various values of h = HS/Hdf. K is the solution of the quadratic equation S2. The K curves all lie between Kdf = 10 and Kmax = 100 cells per microliter. Each curve rises with increasing I as proliferation of K is stimulated, and K stabilises above the I half-saturation level of 2.5. The maximum of K decreases as h decreases. (PDF) [file pone.0049492.s002.pdf]

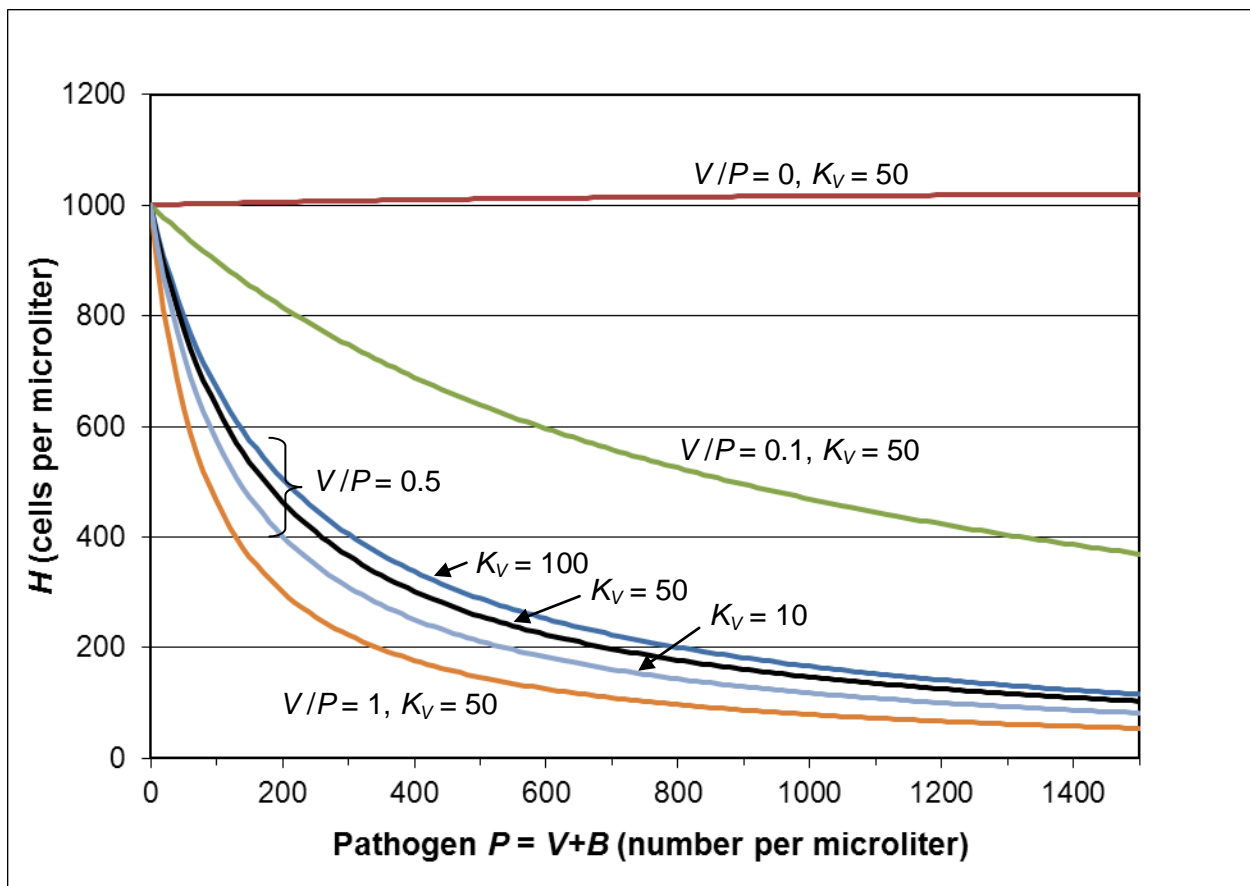

Supplement: Figure S2 — CD4+ helper T cell dependence on pathogen level. CD4+ helper T cell level HS in the equilibrium state is plotted against the combined pathogen level P = V (HIV)+B (MTB). Various curves are shown for different values of HIV-specific CD8+ killer T cell level KV and HIV proportion V/P. The upper curve (V/P = 0, KV = 50) is almost constant since there is no loss of HS to infection and rises slightly with B due to increased proliferation. The curves with V/P>0 decrease with pathogen level even for a small proportion of V. Three curves are shown keeping V/P constant but varying KV from Kdf to Kmax. Although there is some dependence on KV (HS decreases as KV decreases) the main dependence is on V, by loss to HIV infection. (PDF) [file pone.0049492.s003.pdf]

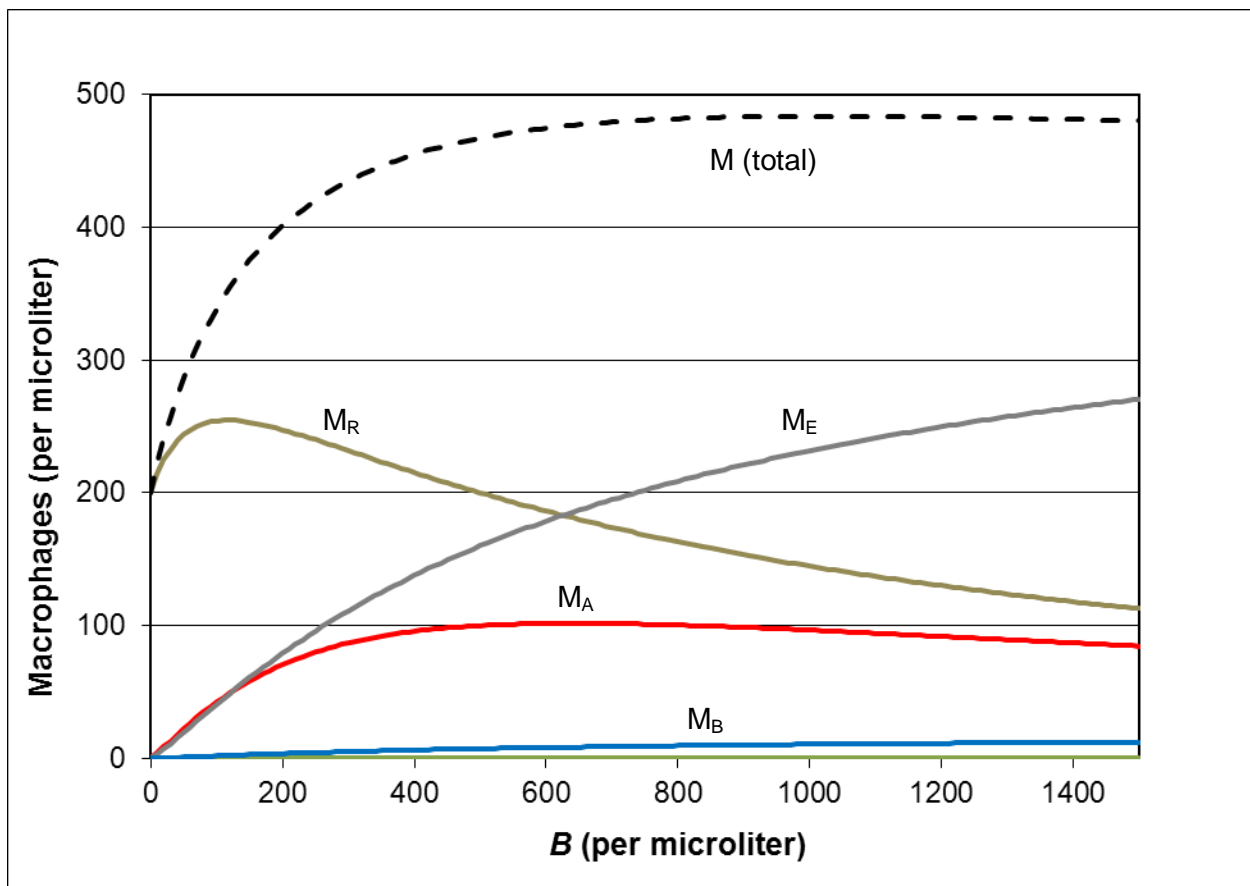

Supplement: Figure S3 — Macrophage populations as functions of MTB level. Equilibrium macrophage levels are plotted against bacterial load B. The dashed line is the total of macrophages in the following states: resting MR, activated MA, MTB-infected eclipse-stage ME and productively infected MB. Total macrophage population increases with B and stabilises above the half-saturation level of B = 500. Resting macrophages rise at first from the disease-free level of 200 but then fall due to loss to infection. Activated macrophages increase and then stabilise above the half-saturation level, also B = 500, but fall slightly at high B. Eclipse-stage and productive-stage populations both rise with B. (PDF) [file pone.0049492.s004.pdf]

**A**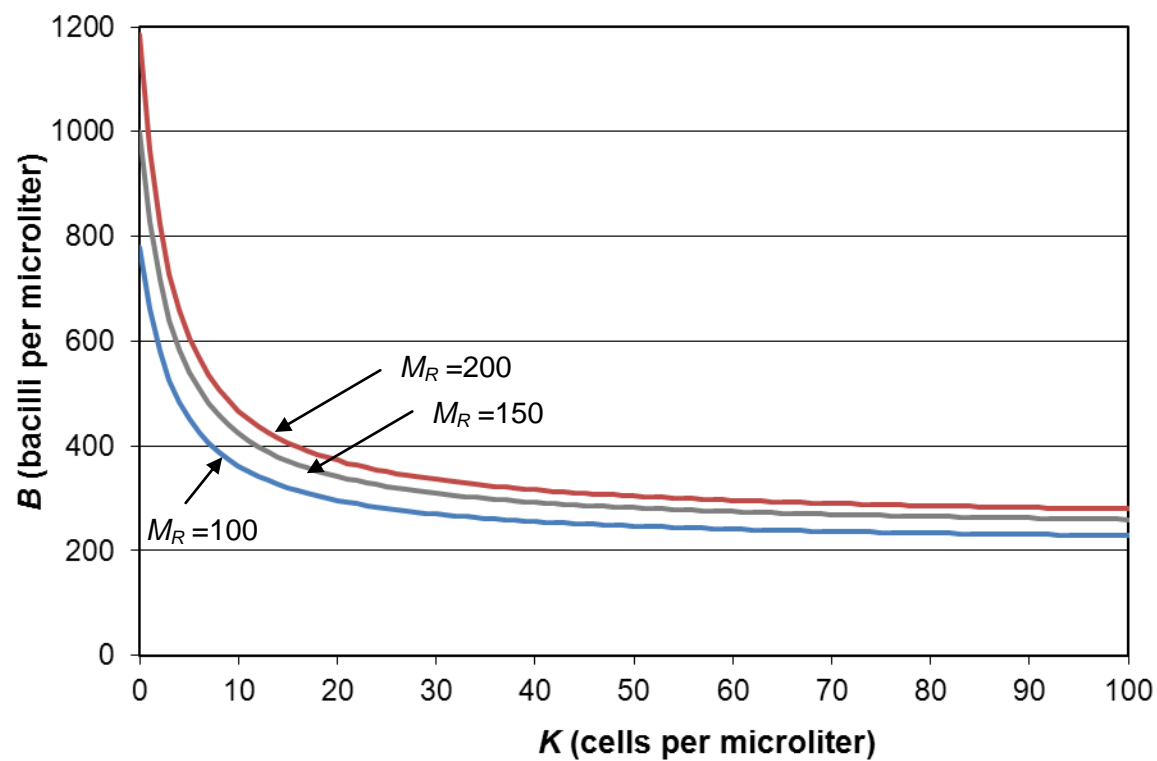**B**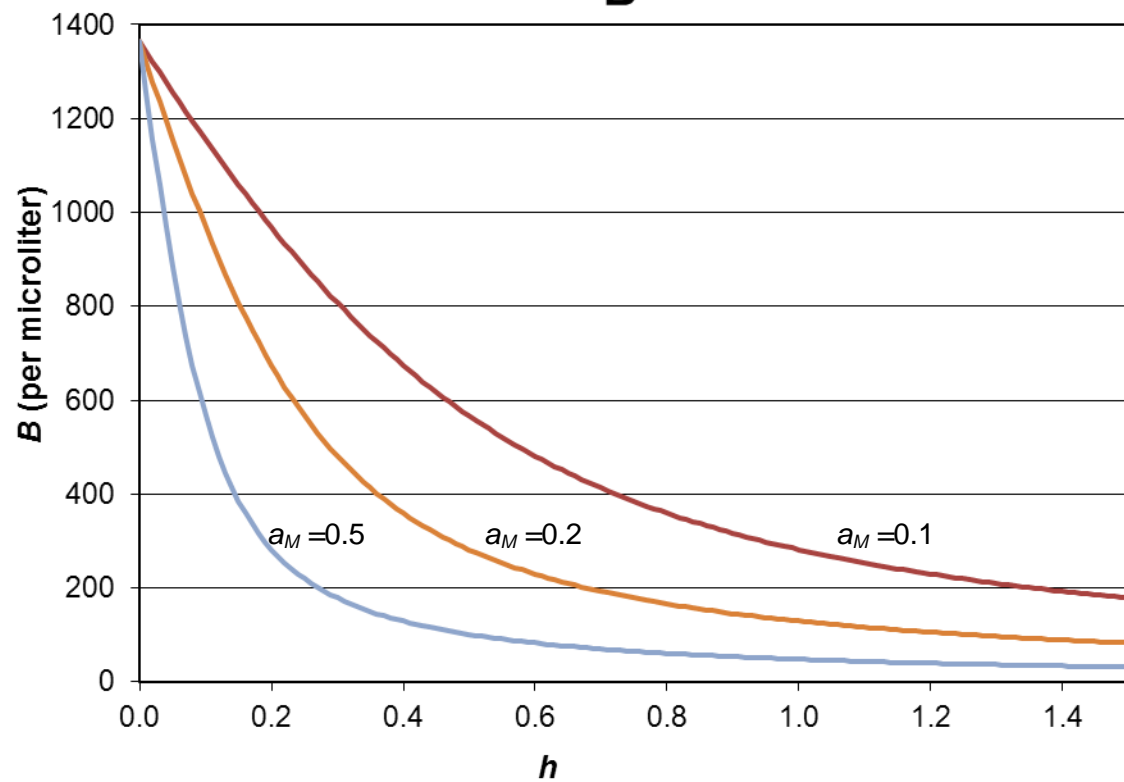

Supplement: Figure S4 — Dependence of MTB load on CD4+ and CD8+ T cell levels. Equilibrium bacterial load B is plotted against MTB-specific CD8+ killer T cell level KB in panel A and against h = HS/Hdf in panel B. Curves are plotted for various values of resting macrophage population MR in panel A and of activation parameter aM in panel B. Panel A shows that B depends strongly on KB in the range 0–20 cells per microliter but does not vary much as KB increases to 100. There is less dependence on MR. Panel B shows that B decreases strongly with h = HS/Hdf and with increasing aM. This is due to the dependence of the activated macrophage population on the product haM. (PDF) [file pone.0049492.s005.pdf]

**A**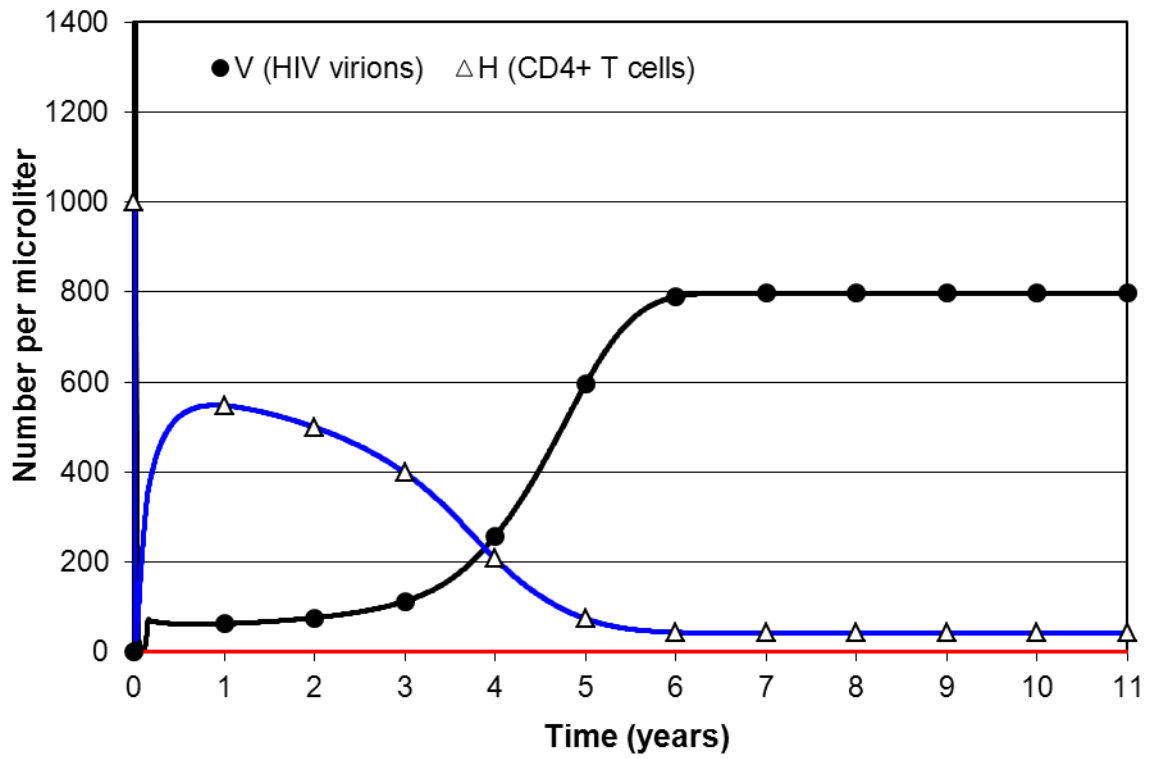**B**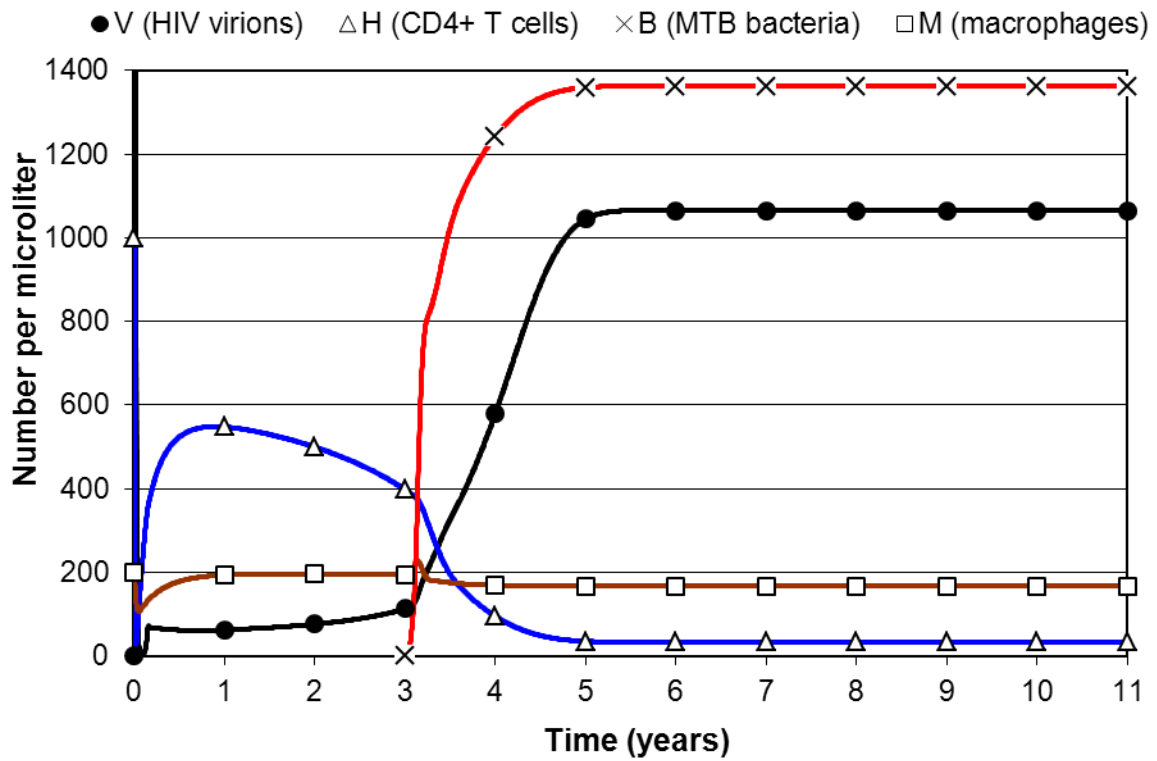

Supplement: Figure S5 — Simulation of HIV-MTB co-infection with reduced model. The graph shows the same scenario as in Figures 2A and 4A, but with the reduced model in which MA, ME, and HE are omitted. The absence of eclipse-stage populations results in shortened time-lags. Panel A shows the HIV-only simulation in which the initial spike in V is much sooner (day 4) and much higher (30 000 mm−3) than for the full model. Panel B (HIV-MTB co-infection) shows that MTB infection results in a sudden rise of bacterial load to its maximum level, which does not model the characteristic slow growth of MTB. (PDF) [file pone.0049492.s006.pdf]

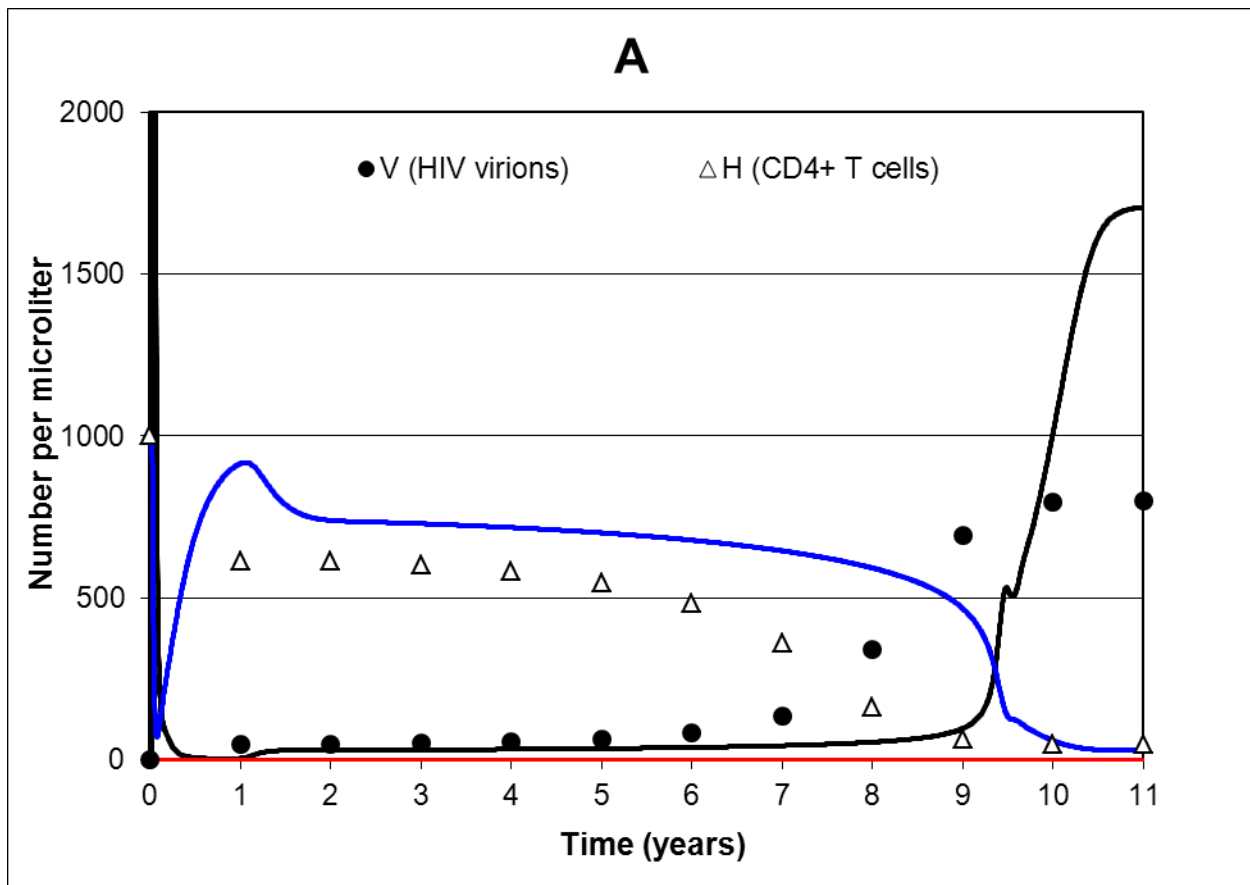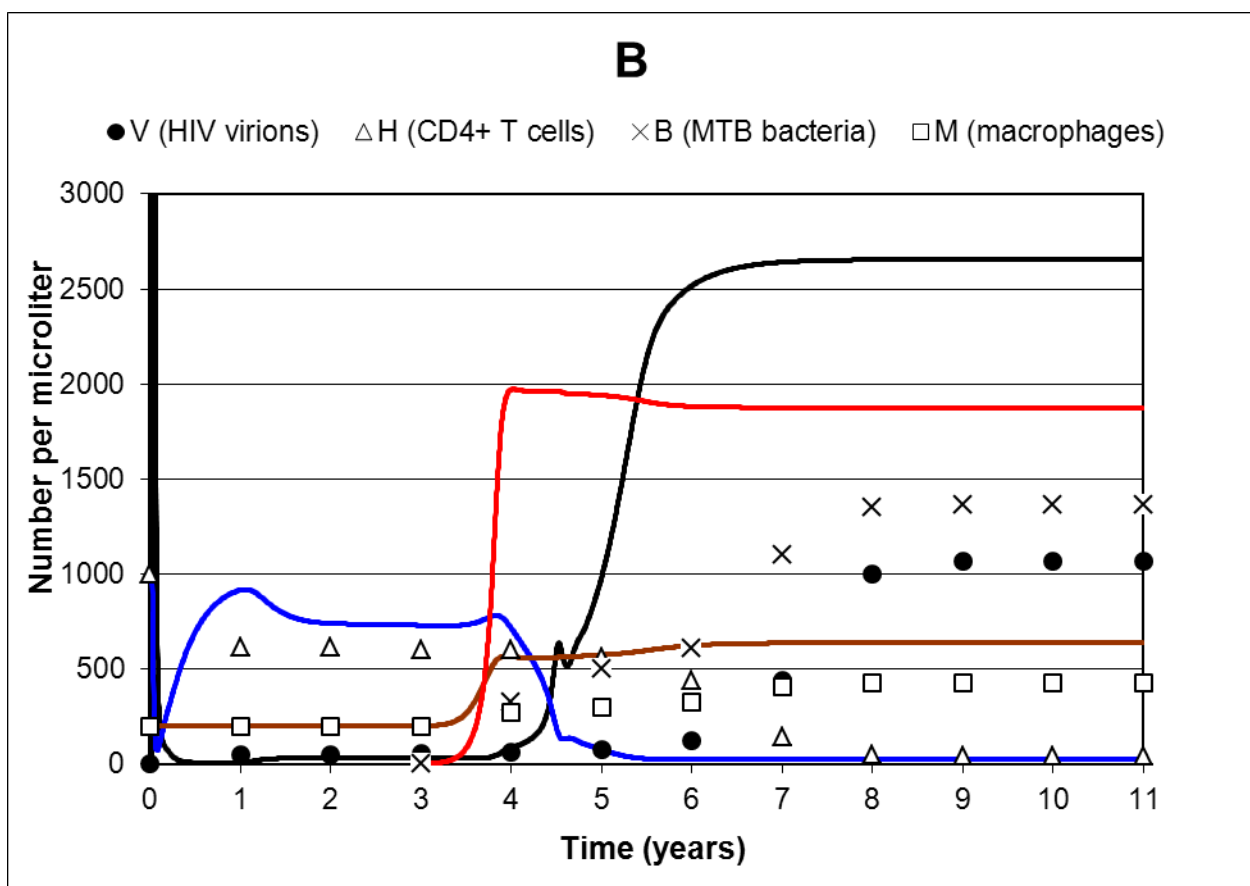

Supplement: Figure S6 — Simulation with full model but without immune response. To show dependence of the system on immune response, the full model is run as for Figures 2A and 4A but without macrophage activation or lysis by CD8+ killer T cells. However, the weak response of ingestion of MTB by resting macrophages is retained otherwise there would be no bacterial growth. Results without immune response are shown by solid lines and with immune response by markers. The simulation is compared in what follows to the full model. Panel A (HIV-only) shows that the initial spike in V is sooner and much higher. Due to initially high viral load, HS falls more at first and consequently V is lower later on since it has fewer target cells to infect, so HS has a higher set-point level. Transition to AIDS is short (6 months) and abrupt. Panel B shows HIV-MTB co-infection. MTB infection leads to a rapid rise in B (since there are neither activated macrophages nor CD8+ T cells to control infection) which triggers an immediate transition to AIDS with much higher viral and bacterial loads than in the model with the immune response. (PDF) [file pone.0049492.s007.pdf]
